# Systematic Two-Hybrid and Comparative Proteomic Analyses Reveal Novel Yeast Pre-mRNA Splicing Factors Connected to Prp19

**DOI:** 10.1371/journal.pone.0016719

**Published:** 2011-02-28

**Authors:** Liping Ren, Janel R. McLean, Tony R. Hazbun, Stanley Fields, Craig Vander Kooi, Melanie D. Ohi, Kathleen L. Gould

**Affiliations:** 1 Howard Hughes Medical Institute, University of Washington, Seattle, Washington, United States of America; 2 Department of Cell and Developmental Biology, Vanderbilt University School of Medicine, University of Washington, Seattle, Washington, United States of America; 3 Department of Genome Sciences and Department of Medicine, University of Washington, Seattle, Washington, United States of America; 4 Department of Molecular and Cellular Biochemistry and Center for Structural Biology, University of Kentucky, Lexington, Kentucky, United States of America; University of South Florida College of Medicine, United States of America

## Abstract

Prp19 is the founding member of the NineTeen Complex, or NTC, which is a spliceosomal subcomplex essential for spliceosome activation. To define Prp19 connectivity and dynamic protein interactions within the spliceosome, we systematically queried the *Saccharomyces cerevisiae* proteome for Prp19 WD40 domain interaction partners by two-hybrid analysis. We report that in addition to *S. cerevisiae* Cwc2, the splicing factor Prp17 binds directly to the Prp19 WD40 domain in a 1∶1 ratio. Prp17 binds simultaneously with Cwc2 indicating that it is part of the core NTC complex. We also find that the previously uncharacterized protein Urn1 (Dre4 in *Schizosaccharomyces pombe*) directly interacts with Prp19, and that Dre4 is conditionally required for pre-mRNA splicing in *S. pombe*. *S. pombe* Dre4 and *S. cerevisiae* Urn1 co-purify U2, U5, and U6 snRNAs and multiple splicing factors, and *dre4Δ* and *urn1Δ* strains display numerous negative genetic interactions with known splicing mutants. The *S. pombe* Prp19-containing Dre4 complex co-purifies three previously uncharacterized proteins that participate in pre-mRNA splicing, likely before spliceosome activation. Our multi-faceted approach has revealed new low abundance splicing factors connected to NTC function, provides evidence for distinct Prp19 containing complexes, and underscores the role of the Prp19 WD40 domain as a splicing scaffold.

## Introduction

The spliceosome is a dynamic ribonucleoprotein complex that catalyzes the removal of introns from pre-mRNA in two discrete steps. It is comprised of five snRNAs (U1, U2, U4, U5, and U6) bound both to intimately associated proteins that form ribonucleoprotein particles (snRNPs) and a host of other conserved protein factors, many whose function are not well understood (reviewed in [1]). Spliceosome assembly occurs in discrete stages. The spliceosome assembly reaction is initiated when the 5′ and 3′ splice sites are recognized by the U1 and U2 snRNPs, respectively, forming complex A. The subsequent engagement of the U5.U4/U6 tri-snRNP to form complex B disrupts U1 binding to the pre-mRNA and triggers unwinding of the U4/U6 snRNA duplex, which is replaced by a U2/U6 snRNA duplex. Further reorganization occurs upon release of the U1 and U4 snRNPs and addition of the Prp19-associated NineTeen Complex (NTC) to form complex B*, marking spliceosomal activation. 5′splice site cleavage and lariat formation then occur in complex C, and finally the 3′ splice site is cleaved, the exons are ligated, and the spliceosome is released from the mRNA product.

Regulation of the structural rearrangements among snRNPs, the NTC, and other proteins is not fully understood but the transition from an inactive to an active spliceosome correlates with stable NTC binding [2], [3], [4], [5]. The NTC promotes new interactions between the U5 and U6 snRNAs with the pre-mRNA, and destabilizes interactions between the U6 snRNA and Sm-like (Lsm) proteins during complex C formation [2], [3]. However, the mechanistic details of the NTC's effects remain unknown.

**Figure 1:**
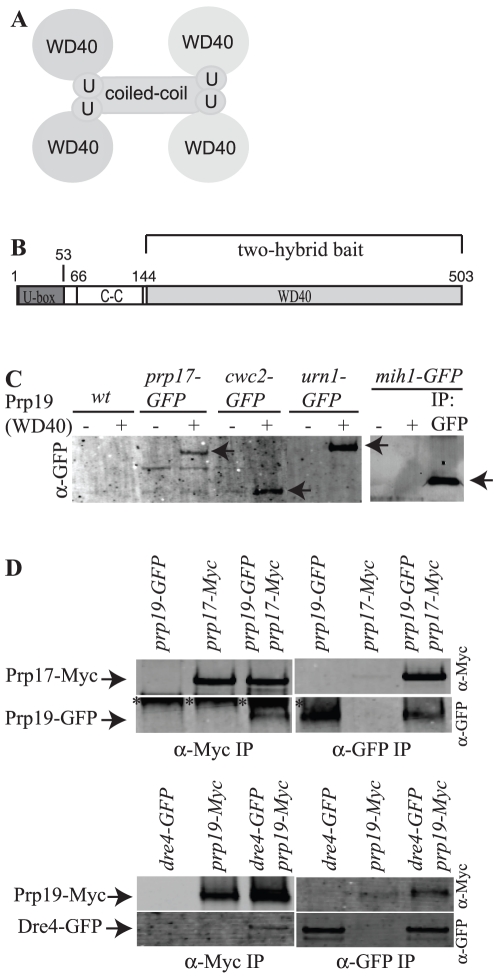
Identification of Prp19 interactors. A) Model of Prp19 architecture. Domains are not drawn to scale. U = U-box. B) Schematic of Prp19 domains drawn to scale. The region used for the two-hybrid screen is indicated. C-C = coiled-coil. C) Ni^2+^-NTA beads alone (−) or with (+) bound Prp19(144–503) were incubated with *S. cerevisiae* protein lysates expressing the indicated GFP-tagged proteins. Bound proteins and anti-GFP immunoprecipitated protein were detected by immunoblotting with anti-GFP antibodies and are indicated with arrows. D) Anti-Myc (left panels) or anti-GFP immunoprecipitates (right panels) from the indicated *S. pombe* strains were blotted with antibodies to the Myc epitope (top panels) or GFP (bottom panels). Asterisks indicate a band recognized by the anti-GFP antibodies non-specifically in anti-Myc antibody immunoprecipitations.

In *Saccharomyces cerevisiae*, the NTC has been purified as a distinct unit composed of about 10 proteins [6], many of which have been identified and are conserved in *Schizosaccharomyces pombe* and human spliceosomal complexes [1], [7], [8], [9], [10]. The namesake of the NTC, Prp19 (also known as *S. cerevisiae* Pso4, human SNEV or NMP200, and Cwf8 in *S. pombe*; hereafter referred to as Prp19 for orthologs in any organism), is a tetrameric protein that oligomerizes through a central coiled-coil domain in an anti-parallel manner [11] (see Figure 1A). Cef1 and Snt309 bind directly to the tetrameric coiled-coil domain [11]. From each end of the tetramerization domain protrudes a dimer of the Prp19 N-terminal U-box domain [12], which confers E3 ubiquitin ligase activity to the protein [13], [14], [15]. Also protruding from each end of Prp19's central stalk are two globular C-terminal WD40 domains. Given that WD40 repeats mediate protein-protein interactions, it is likely that each WD40 repeat interacts with other spliceosome components. However, only one NTC binding partner, Cwc2, has been identified for this domain [8], [16].

Although first identified in *S. cerevisiae* based on its role in pre-mRNA splicing, Prp19 has been implicated in other processes including DNA repair [17], [18], recombination [19], sporulation [20], nuclear matrix structure, [21], and siRNA-mediated centromeric transcriptional silencing [22]. Also, NTC components associate with activation-induced deaminase [23]. Presently, it is unclear whether all these reported activities reflect splicing dependent or independent functions and whether Prp19 might be a multi-functional protein that interacts with distinct groups of proteins to carry out different functions. Certainly, the modular nature of its architecture might allow it to interact with both splicing and non-splicing factors.

In an attempt to identify the full complement of proteins capable of interacting with the WD40 domain of Prp19, we performed a global yeast two-hybrid screen using the *S. cerevisiae* Prp19 WD40 domain as bait and went on to investigate whether positives in the screen directly interacted with this domain. In addition to its known interaction with Cwc2 [8], we found that Prp19 binds directly to the splicing factor, Prp17, and the uncharacterized protein, Urn1. Interactions among NTC components are conserved between *S. pombe* and *S. cerevisiae* [8], [11], [24] and we used both yeast species here to examine biochemical properties, genetic interactions, and functions involving Prp17 and Urn1. For clarity, we will frequently refer to *S. cerevisiae* proteins with the prefix *Sc* and *S. pombe* proteins with the prefix *Sp*. From both yeasts, *Sc*Urn1/*Sp*Dre4 purifications contain multiple known splicing factors, U2, U5, and U6 snRNAs and *Sp*Dre4 is conditionally required for pre-mRNA splicing. Additionally, *Sp*Dre4 co-purified four previously uncharacterized proteins essential for, or impact, pre-mRNA splicing, two of which are apparently absent in *S. cerevisiae*. Thus, our combinatorial approaches led to the discovery of new splicing factors connected to the NTC and highlight a major function of the Prp19 WD40 domains as a scaffold for splicing proteins.

## Materials and Methods

### Yeast two hybrid analyses

Sequences encoding amino acids 146–503 of *Sc*Prp19 were subcloned into the pOBD2 vector. The genome-wide two-hybrid screen using this bait was performed with robotics as described previously [25], [26], [27]. Other yeast two-hybrid assays were done as described using *S. cerevisiae* strain PJ69-4A and the pGBT9 and pGAD vectors [28]. ß-galactosidase reporter enzyme activity in the two-hybrid strains was measured using the Galacto-Star™ chemiluminescent reporter assay system according to the manufacturer's instructions (Applied Biosystems, Foster City, California), except that cells were lysed by glass bead disruption. Each sample was measured in triplicate. Reporter assays were recorded on a Multi-Detection Microplate Reader (Bio-TEK Instruments, Inc. Vermont, USA).

### Strains and media

*S. pombe* strains (Table S1) were grown in yeast extract medium or minimal medium with appropriate supplements [29]. Transformations were performed by the lithium acetate method [30], [31]. Epitope tagged strains were constructed as described previously [32], [33] so that open reading frames were tagged at the 3′end of endogenous loci with the GFP-Kan^R^, TAP-Kan^R^, V5_3_-Kan^R^, FLAG_3_-Kan^R^, or a linker-TAP-Kan^R^ cassette. Appropriate tagging was confirmed by PCR and immunoblotting. Strain construction and tetrad analysis were accomplished through standard methods. For spore germination experiments, mating colonies were grown in glutamate medium over night at 25°C, washed with 30% ethanol, washed with water, and then incubated in minimal medium at 32°C in the absence of uracil. *S. cerevisiae* strains used in this study are listed in Table S1. They were grown either in synthetic minimal medium with the appropriate nutritional supplements or yeast extract-peptone-dextrose [34].

### Molecular Biology Techniques

All plasmid constructions were performed by standard molecular biology techniques. All DNA oligonucleotides were synthesized by Integrated DNA Technologies, Inc. (Iowa). All sequences were PCR amplified with Taq-Plus Precision (Stratagene) according to manufacturer's protocol. Site-directed mutagenesis was carried out using Quickchange (Stratagene) according to manufacturer protocols.

### Specimen preparation, electron microscopy, and image processing

Uranyl formate (0.7% w/v) was used for negative staining as described [35]. Images of samples were recorded using a Tecnai T12 electron microscope (FEI) equipped with a LaB_6_ filament and operated at an acceleration voltage of 120 kV. Images were taken under low-dose conditions at a magnification of 67,000X using a defocus value of −1.5 µm and recorded on DITABIS digital imaging plates (Pforzheim, Germany). The plates were scanned on a DITABIS micron scanner (Pforzheim, Germany), converted to mixed raster content (mrc) format, and binned by a factor of 2 yielding final images with 4.48 Å/pixel.

Particles (7,963) were selected interactively using WEB, and windowed into 120×120 pixel images (4.48 Å/pixel). The images were rotationally and translationally aligned and subjected to 10 cycles of multi-reference alignment and K-means classification into 25 classes using the processing package SPIDER [36].

### Analytical Ultracentrifugation

Sedimentation velocity experiments were run at 30,000 RPM (22°C) on an Optima XLI (Beckman-Coulter, Fullerton, CA), with a 4-hole An60Ti rotor using double sector cells with charcoal-filled Epon centerpieces (path length 1.2 cm) and sapphire windows. The velocity scans were analyzed with the program Sedfit (version 8.7) [37] using 250 scans collected approximately 2 min apart. Size distributions were determined for a confidence level of p = 0.95, a resolution of n = 200, and sedimentation coefficients between 0.3 and 35 s. SVAU experiments generally provide only a predicted molecular weight and the shape of the molecule (i.e. indicated by the frictional ratio) is an important element in this calculation. When there is a mixture of two differently shaped molecules in the sample, as is likely the case for MBP and MBP-Prp17 (Table 1), the predicted molecular weight may be smaller than expected.

**Table 1:** Sedimentation velocity analytical centrifugation data summary.

	S values	Predicted MW (kDa)	Frictional Ratio	r.m.s.d.
**Prp19:**			1.8	0.0057
Peak 1	6.10 (85%)	211.6		
Peak 2	2.13 (14%)	41.7		
**His_6_-Urn1**			1.5	0.0063
Peak 1	3.6 (95%)	58.5		
**Prp19:His_6_-Urn1**			1.63	0.0050
(1∶1 molar concentration)				
Peak 1	8.97 (38%)	295.8		
Peak 2	3.56 (42.7%)	62.9		
**Prp19:His_6_-Urn1**			1.5	0.0058
(1∶2.5 molar concentration)				
Peak 1	9.03 (22.4%)	312.5		
Peak 2	3.45 (69.8%)	56.5		
**MBP-Prp17**			1.75	0.0057
Peak 1	6.54 (7.1%)	190.1		
Peak 2	3.75 (50.5%)	85.5		
Peak 3	1.99 (23.5%)	32.3		

### RNA isolation and detection

2-liters of 4X YE or 8 liters of YPD cultures of *S. pombe* and S. *cerevisiae* TAP strains, respectively, were grown to log phase and the tagged proteins isolated as described [38]. Associated RNAs were isolated from the final elute and total RNA was extracted from 6×10^8^ cells using hot acid phenol [39]. snRNAs were isolated from wild-type cells using an anti-snRNA cap (antitrimethylguanosine [m3G]) immunoprecipitation. The snRNA samples and eluted RNAs from TAP samples were resolved on a 6% Tris-Borate-EDTA–Urea gel (Invitrogen), transferred to a Duralon-UV membrane (Stratagene), UV cross-linked (UVC500 crosslinker –energy setting 700; Amersham Biosciences), and detected by using γ-^32^P ATP (PerkinElmer) labeled oligonucleotides complementary to *S. pombe* U1 (SPU1), U2 (U2B), U4 (SPU4), U5 (YU5), and the exon of U6 (U6E). Blots were exposed to PhosphorImager screens and visualized by using a Typhoon 9200 (Molecular Dynamics). Reverse transcription-PCR was performed with the OneStep RT-PCR kit (Qiagen GmbH, Hilden, Germany) according to the manufacturer's directions. Two hundred nanograms of RNA were used for each reaction. Oligonucleotides flanking the longest intron of *S. pombe prp19^+^* were used to detect unspliced and spliced *prp19* RNAs. RT-PCR products were resolved on 2.5% Nusieve agarose gels (CBS, Rockland, ME).

### Expression of recombinant fusion proteins

Amino acids 144–503 of *Sc*Prp19 were subcloned into the pET15b vector. Fragments of other cDNAs and *ScPRP17* were cloned into pMAL-c2X. *ScURN1* and full-length *ScPRP19* were subcloned into pETDuet-1. Fusion proteins were produced in BL21 bacterial cells and purified from bacterial lysates using Ni-NTA agarose (Qiagen) or amylose beads (New England Biolabs), as specified by the manufacturers and washed in bead binding buffer (20 mM Tris pH 7.5, 100 mM NaCl, 10% glycerol, 10 mM ZnSO_4_, 1 mM imidazole). For AU analysis, *Sc*Prp19 and *Sc*Urn1 were further purified by heparin agarose affinity and gel filtration. For *in vitro* binding assays, recombinant proteins were incubated together for one hour at 4°C and washed extensively prior to analysis by SDS-PAGE and coomassie staining.

### Immunoprecipitations, immunoblotting, and sucrose gradients

Cell pellets were frozen in a dry ice/ethanol bath and lysed by bead disruption in NP-40 lysis buffer under either native or denaturing conditions as previously described [40]. For pulldowns by His_6_-Prp19(144–503), ∼1 µg yeast protein lysate was mixed with 50 µl (1∶1) slurry of beads that were incubated first for 1 hr at 4°C with a solution of 1 µg/ml BSA, washed once with NP-40 buffer with 5 mM imidazole, twice with bead binding buffer and then either beads alone or with recombinant fusion protein. After mixing for 1 hr at 4°C, the beads were collected and washed 3 times with bead binding buffer with 5 mM imidazole before analysis by immunoblotting.

Proteins were immunoprecipitated from various amounts of protein lysates using anti-FLAG (M2, Sigma-Aldrich), anti-HA (12CA5), anti-V5 (Invitrogen), anti-GFP (Roche), rabbit IgG (for TAP), or anti-Myc (9E10) followed by Protein G, Protein A, or IgG Sepharose beads (GE Healthcare).

For immunoblotting, proteins were resolved by 10% SDS-PAGE, transferred by electroblotting to a polyvinylidene difluoride membrane (Immobilon P; Millipore Corp., Bedford, Mass) and incubated with the set of primary antibodies indicated at 1 µg/ml. Primary antibodies were detected with secondary antibodies coupled to Alexa Fluor 680 (Invitrogen, CA) or IRDye800 (LI-COR) and visualized using an Odyssey Infrared Imaging System (LI-COR Biosciences, NE). Quantifications of protein intensities were performed using Odyssey (LI-COR, NE) version 1.2.

A 200-µl volume corresponding to 20% of the isolated TAP complexes was layered onto a 10 to 30% sucrose gradient and centrifuged at 28,000 rpm for 18 h in a SW50Ti rotor. Fractions from these gradients were collected, mixed with sample buffer, and resolved by SDS-PAGE. Parallel gradients were run; these contained thyroglobulin (19S) and catalase (11.35S) (HWM Standards; Pharmacia) as sedimentation markers.

### TAP and MS analysis

Proteins were purified by TAP as described [38] and subjected to mass spectrometric analysis as previously detailed [41], [42]. RAW files were converted to DTA or MZML files using Scansifter (software developed in-house at the Vanderbilt University Medical Center). Spectra with less than 6 peaks were excluded from our analysis. The *S. pombe* database (http://www.sanger.ac.uk) was searched using the SEQUEST algorithm, and results were processed using the CHIPS program (jointly developed by Vanderbilt University Mass Spectrometry Research Center and University of Arizona). Filter settings for peptides were: Xcorr≥1.8 for singly charged; Xcorr≥2.5 for doubly charged; Xcorr≥3.3 for triply charged. The *S. cerevisae* database (http://www.yeastgenome.org) was searched using Myrimatch [43] and analyzed in IDPicker 2.4.0 [44], [45] using the following filters: max. FDR per result 0.05, max. ambiguous IDs per result 2, min. peptide length per result 5, min. distinct peptides per protein 2, min. additional peptides per protein group 1, indistinct modifications M 15.994 C 57.05. Parsimony rules were applied to generate a minimal list of proteins to explain all of the peptides that passed our entry criteria. Contaminant proteins were included in both databases and all sequences were reversed and concatenated to allow estimation of false discovery rates (10186 entries for *S. pombe* and 13580 entries for *S. cerevisae*).

Other Methods are Described in Methods S1 in Supporting Information.

## Results

### Identification of Prp19 WD40 binding partners

To identify binding partners of the atypical Prp19 WD40 domain [16], we performed a genome-wide two-hybrid screen in duplicate using as bait amino acids 146–503 of *S. cerevisiae* Prp19 fused to the Gal4 DNA-binding domain (Figure 1B) and an array of ∼6000 yeast strains expressing each *S. cerevisiae* ORF fused to the Gal4 activation domain [25], [26]. Positives common to unrelated screens and positives detected in only one of the two screens were not pursued further. Eight potential interactors were identified in both screens: Cwc2, Prp17/Cdc40 (hereafter referred to as Prp17 for both the *S. pombe* and *S. cerevisiae* orthologs), Urn1, Mih1, YOR314w, Uba3, YPL257w, and Ufd4. Cwc2 was expected to be a positive hit in the screen because we previously showed that it is a splicing factor that binds directly to the Prp19 WD40 domain [8], [16] and is conserved as Cwf2 in *S. pombe* [24]. Prp17 and Urn1 had been detected in a previous two-hybrid screen that had used full-length Prp19 as bait [46]. Prp17 is a splicing factor [47], [48], and Urn1 (Dre4 in *S. pombe* (Figure S1)) is an uncharacterized, non-essential protein that co-purifies with the spliceosome [5]. Mih1 is a Cdc25 phosphatase family member involved in cell cycle control [49]. YPL257w is an uncharacterized ORF of unknown function and unknown localization and YOR314w is a dubious ORF. Ufd4 is an E3 ubiquitin ligase [50] and Uba3 is one of two subunits comprising the Nedd8 activating enzyme [51].

Cwc2-GFP, Prp17-GFP, and Urn1-GFP, but not other GFP-tagged hits, were pulled down from *S. cerevisiae* cell lysates by bacterially expressed and purified His_6_-Prp19(144–503) (Figure 1C and data not shown). To determine whether the two new stable associations were conserved throughout evolution, we tested whether the *S. pombe* homologs of Prp17 and Urn1 would interact with Prp19. Indeed, epitope-tagged versions of *S. pombe* Prp17 and *S. pombe* Dre4 co-immunoprecipitated *S. pombe* Prp19 and vice-versa (Figure 1D). We did not detect association of the remaining five two-hybrid positives with Prp19 using these biochemical approaches (Figure 1C and data not shown) and did not investigate them further in this study.

### Characterization of the Prp17-Prp19 interaction

**Figure 2:**
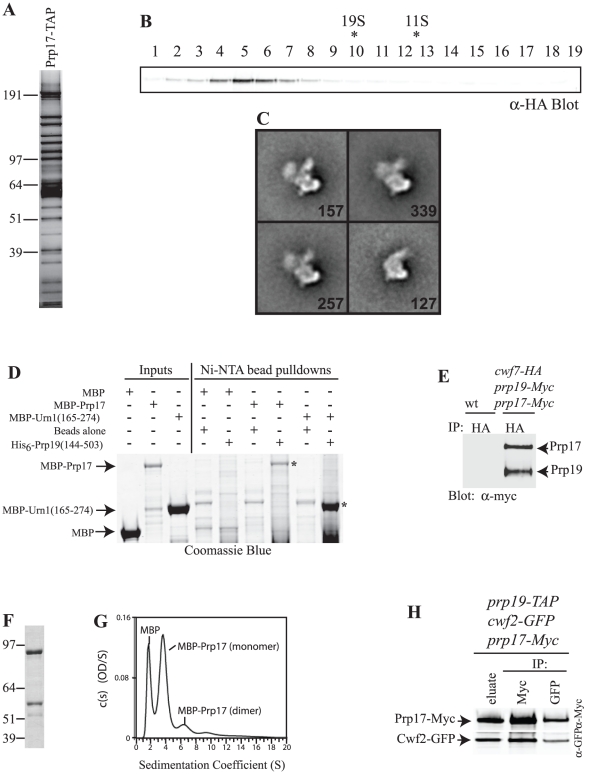
Characterization of Prp19-Prp17 interaction. A) A fraction of the SpPrp17-TAP eluate was analyzed by silver staining. Positions of markers are indicated. B) *Sp*Prp17-HA_3_-TAP eluate was resolved on a 10 to 30% sucrose gradient, and fractions were collected from the bottom (fraction1). These were resolved by SDS-PAGE and immunoblotted with anti-HA to detect the migration of Prp17. Migration of of catalase (11.3S) and thyroglobulin (19S) collected from parallel gradients is indicated with asterisks. C) Four representative class averages of *Sp*Prp17-TAP particles in negative stain. The number of particles in each projection average is shown in the lower right corner of each average. Side length of individual panels is 537.6 Å. D) Purified and soluble MBP, MBP-*Sc*Urn(165–274), or MBP-*Sc*Prp17 (Inputs) were incubated with Ni-NTA beads alone or Ni-NTA beads coated with His_6_-*Sc*Prp19(144–503). Proteins bound to the beads after washing were detected by Coomassie blue staining. Asterisks indicate MBP-*Sc*Prp17 and MBP-*Sc*Urn1 fragments pulled down by the ScPrp19 WD40 domain. The Ni-NTA beads alone did not pull down MBP or MBP fusion proteins, but did pull down some non-specifically binding bacterial proteins. E) An anti-HA immunoprecipitate from *S. pombe cwf7-HA prp19-Myc_13_ prp17-Myc_13_* cells was blotted for the presence of Myc-tagged proteins. Bands were quantified on an Odyssey instrument. F) Coomassie stained gel of purified MBP-*Sc*Prp17 produced in *E. coli*. Note the degradation bands. G) Continuous size distribution analysis of sedimentation velocity data of MBP-*Sc*Prp17. AU experiments were conducted at 22°C at a speed of 30,000 rpm and concentration profiles measured at 280 nm. H) *Sp*Prp19-TAP complex was isolated from a *S. pombe prp19-TAP cwf2-GFP prp17-Myc_13_* strain and a portion of the eluate was probed for the presence of *Sp*Prp17 and *Sp*Cwf2. The remainder of the eluate was divided in half. One half was immunoprecipitated with anti-Myc and the other with anti-GFP and then each immunoprecipitate was immunoblotted with anti-GFP or anti-Myc antibodies.

**Figure 3:**
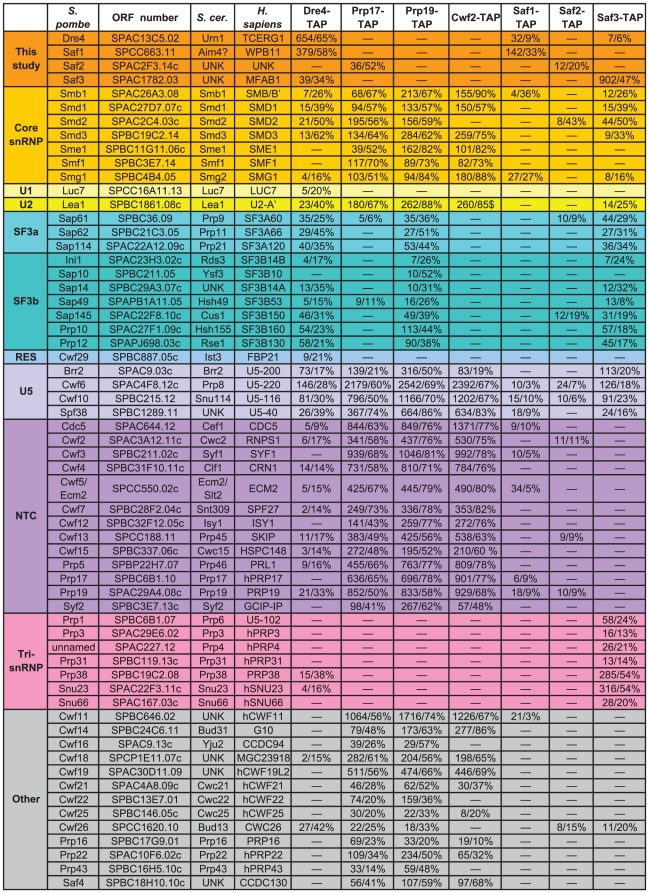
Mass spectrometric analysis of *S. pombe* splicing associated factors. Proteins are categorized by sub-complex with the number of spectral counts and percent sequence coverage provided. Components present at less than 5% sequence coverage or with less than five distinct peptides were not included in the compilation of splicing factors based on subcomplexes. Full analyses of mass spectrometric data are provided in Tables S2 and S3. UNK = unknown.

Prp17 has been identified in isolations of the splicing apparatus from multiple organisms [1] including yeasts [24], [52], and *Sc*Prp17 co-purifies the U2, U5 and U6 snRNAs [48]. To determine the *Sp*Prp17 associated splicing factors, it was tagged with the TAP or HA_3_-TAP epitope and purified. Proteins present in *Sp*Prp17-TAP complexes were visualized by silver staining (Figure 2A) and identified by 2D-LC-MS/MS (Figure 3 and Table S2). The compilation of associated splicing factors was compared to that of other *S. pombe* NTC components such as *Sp*Cdc5 [24], *Sp*Cwf2 and *Sp*Prp19 (Figure 2A, Figure 3, and Table S2), which all co-purified primarily U2, U5 and NTC components. The *Sp*Prp17-HA_3_-TAP eluate sedimented on a sucrose gradient with a single peak of comparable size to the *Sp*Cdc5-TAP complex (Figure 2B) [24], [53] indicating that, like Cdc5 [53], all Prp17 is associated with this complex. Furthermore, the complex purified by *Sp*Prp17-TAP appears by electron microscopy to be very similar in size and homogeneity (Figure 2C and data not shown) to that purified by Cdc5 [54]. We conclude that *Sp*Prp17 is a stable component of the NTC.

The domain of *Sc*Prp17 that binds *Sc*Prp19 was defined by directed two-hybrid analysis. *Sc*Prp17 N-terminal sequences, but not its WD40 repeats, conferred the ability to interact with *Sc*Prp19 (Figure S2A). The Prp19-interacting domain was then fused to Maltose Binding Protein (MBP), produced in bacteria and purified. MBP-*Sc*Prp17(1–140), but not MBP, was able to specifically bind Ni^2+^ beads coated with His_6_-*Sc*Prp19(144–503) (Figure 2D). Thus, like *Sc*Cwc2, *Sc*Prp17 can bind directly to the *Sc*Prp19 WD40 region.

The tetrameric architecture of Prp19 [11] could allow multiple copies of Prp17 to be present in the NTC if each Prp19 WD40 contains a Prp17 binding site. Because it is known that one molecule of *Sp*Cwf7 binds tetrameric *Sp*Prp19 [11], it is possible to determine the precise copy number of other NTC components using quantitative immunoblot analysis. Complexes were purified from *S. pombe cwf7-HA_3_ prp19-Myc_13_ prp17-Myc_13_* cells through anti-HA immunoprecipitation, and the Myc-tagged proteins were detected by immunoblotting (Figure 2E). The 1.05∶1 ratio of Myc-tagged proteins, determined by quantitation on an Odyssey instrument, shows that *Sp*Prp17 is present in at least equivalent amounts to *Sp*Prp19. To provide insight into the possible binding arrangement of Prp17 relative to Prp19, *Sc*Prp17 was produced in *E. coli* as a fusion with MBP, purified (Figure 2F), and subjected to analytical ultracentrifugation. MBP-*Sc*Prp17 (predicted kDa of 94) was primarily monomeric (Figure 2G and Table 1), a result consistent with the possibility that in cells, each Prp19 WD40 repeat is bound by a monomer of Prp17.

Two molecules of monomeric *Sc*Cwc2 bind to each *Sc*Prp19 tetramer through the WD40 domain [16]. To test whether Prp17 and Cwf2 could exist in the same Prp19 complex, *S. pombe* Prp19-TAP complexes were isolated from *prp19-TAP cwf2-GFP prp17-Myc* cells and the TAP eluate was split into two portions to probe the ability of *Sp*Cwf2 to co-immunoprecipitate *Sp*Prp17 and vice-versa. Their co-immunoprecipitation (Figure 2H) indicates that *Sp*Prp17 and *Sp*Cwf2 can bind *Sp*Prp19 simultaneously.

### Characterization of the Urn1/Dre4-Prp19 interaction

**Figure 4:**
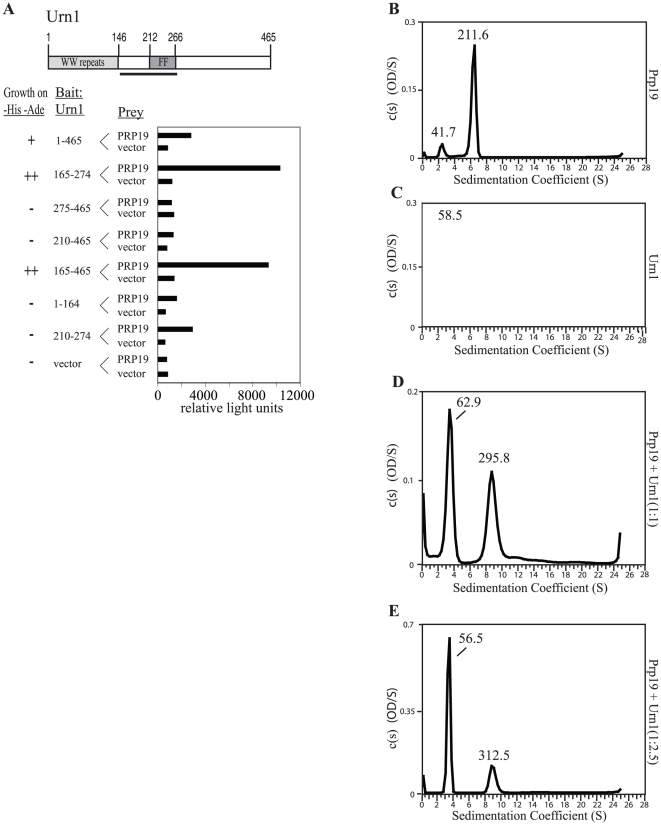
Characterization of Prp19-*Sc*Urn1/*Sp*Dre4 interaction. A) *S. cerevisiae* strain pJ69-4A was cotransformed with bait plasmids expressing the indicated regions of *ScURN1*. + indicate growth and – denotes no growth on selective media. ß-galactosidase activity (represented by relative light units) of the transformants is plotted in the right panels. (B–E) Continuous size distribution analysis of sedimentation velocity data of *Sc*Prp19, His_6_-*Sc*Urn1, and *Sc*Prp19:His_6_-*Sc*Urn1. Calculated c(*s*) is plotted versus sedimentation coefficients (s) for (B) *Sc*Prp19, (C) His_6_-*Sc*Urn1, (D) *Sc*Prp19:His_6_-*Sc*Urn1 in a 1∶1 molar ratio and (E) *Sc*Prp19:His_6_-*Sc*Urn1 in a 1∶2.5 molar ratio. Each s peak is labeled with predicted molecular mass (kDa). *Sc*Prp19 concentrations were constant, 10 mM, with His_6_-*Sc*Urn1 concentrations varied to the indicated molar ratio. AU experiments were conducted at 22°C at a speed of 30,000 rpm and concentration profiles measured at 280 nm.

To define the physical association of *Sc*Urn1 with *Sc*Prp19, we first refined the interaction domains by directed two-hybrid analysis. *Sc*Urn1 sequences including the FF domain, the structure of which has been determined [55], were sufficient for Prp19 interaction (Figure 4A). The Prp19-interacting domain was fused to MBP, produced in *E. coli*, and purified. MBP-*Sc*Urn1(165–274), but not MBP, was able to specifically bind Ni^2+^ beads coated with His_6_-*Sc*Prp19(144–503) (Figure 2D).

To investigate the nature of this association further, full length *S. cerevisiae* Prp19 and His_6_-*Sc*Urn1 were co-expressed in *E. coli*, purified, and analyzed using velocity sedimentation analytical ultracentrifugation. *Sc*Prp19 (mass, 58.6 kDa) exists predominantly as an extended tetramer (s = 6.1, 85%) (Figure 4B, Table 1), as previously demonstrated [16]. His_6_-*Sc*Urn1 (mass, 55.1 kDa) was found to exist in a monomeric state (s = 3.6, 95%) (Figure 4C, Table 1). Mixing the proteins in a 1∶1 ratio produced a discrete higher order species (s = 9.04, 38%) with a predicted molecular weight (mass, 296 kDa) consistent with a 4∶1 complex of Prp19:Urn1 (mass, 281 kDa) (Figure 4D, Table 1). Increasing the concentration of Urn1 more than two-fold resulted in only a slight increase in apparent molecular weight of the complex (Figure 4E, Table 1). These data confirm that Prp19 and Urn1 directly associate with one another, with a single molecule of Urn1 able to stably bind the Prp19 tetramer.

### *S. cerevisiae* Urn1 and *S. pombe* Dre4 are splicing factors

**Figure 5:**
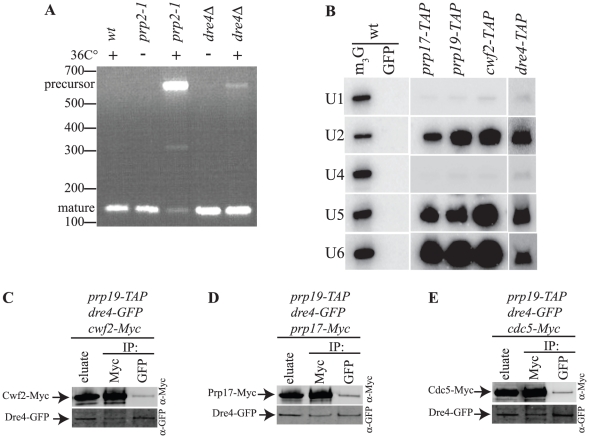
*Sc*Urn1/*Sp*Dre4 is involved in pre-mRNA splicing. A) RNA was purified from the indicated *S. pombe* strains grown at 25°C(−) or shifted to 36°C (+) for 4 hours. RT-PCR reactions were performed using oligonucleotides that flank the long intron within the *prp19* mRNA. PCR products were separated on 3% Nusieve gels and detected with ethidium-bromide and UV imaging. B) Northern analysis of RNA isolated from an anti-cap or anti-GFP immunoprecipitate from wild-type cells, or RNA isolated from the indicated TAP purifications. Each RNA sample was probed for the presence of the U1, U2, U4, U5, and U6 snRNAs. C–E) Prp19-TAP complexes were isolated from the indicated *S. pombe* strains and a portion of the eluates was probed for the presence of the indicated proteins. The remainder of the eluates were divided in half. One half was immunoprecipitated with anti-Myc and the other with anti-GFP and then each immunoprecipitate was immunoblotted with anti-GFP or anti-Myc antibodies.

While Prp17 and *Sc*Cwc2/*Sp*Cwf2 are known splicing factors, a role for *Sc*Urn1/*Sp*Dre4 in pre-mRNA splicing has not been described previously. We obtained multiple lines of evidence that *Sc*Urn1/*Sp*Dre4 impacts this process. First, *S. pombe dre4Δ* cells are viable but temperature-sensitive for growth (Figure S2B; [56]). At the non-permissive temperature of 36°C, they accumulated *prp19* pre-mRNA, indicative of defective pre-mRNA splicing, whereas wild type cells did not (Figure 5A). Second, like *prp17Δ, dre4Δ* is synthetically lethal with the *cdc5-120* splicing mutation at 25°C; in 12 and 11 tetrads, respectively, no viable Ura+ Ts recombinants were obtained. Similarly, *S. cerevisiae urn1Δ* has been reported to interact negatively with a variety of splicing mutations in global synthetic genetic array screens [57], [58]. Third, *Sp*Dre4 amino acids 1–300, which contain the WW and FF domains (Prp19-interacting region), were sufficient to rescue the *S. pombe dre4Δ* strain at 36°C whereas a truncation expressing only the WW domain (amino acids 1–183) very weakly supported growth and failed to promote wild-type morphology (Figure S2C and data not shown). These data suggest that interaction with Prp19 is critical for *Sp*Dre4 function.

**Figure 6:**
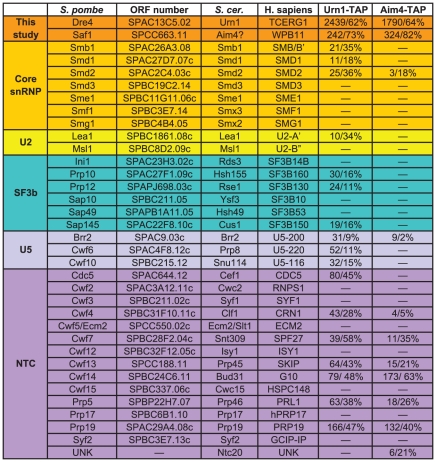
Mass spectrometric analysis of *S. cerevisiae* splicing associated factors. Proteins are categorized by sub-complex with the number of spectral counts and percent sequence coverage provided. Components present at less than 5% sequence coverage or with less than five distinct peptides were not included in the compilation of splicing factors based on subcomplexes. Full analyses of mass spectrometric data are provided in Tables S4 and S5. UNK = unknown.

Sequences encoding a TAP or HA_3_-TAP epitope were added to the 3′end of the *dre4^+^* open reading frame to enable *Sp*Dre4 interacting proteins to be purified and identified by mass spectrometry (Figure 3 and Table S3). *Sp*Dre4-TAP co-purified a large number of splicing factors in addition to *Sp*Prp19 including components of the U2 and U5 snRNPs and the NTC (Figure 3). Proteins known to be involved in other cellular processes were not identified to any significant extent although there was significant background typical of low abundance proteins (Table S3). Indeed, the *Sp*Dre4-TAP complex was not abundant enough to be visualized following sucrose gradient sedimentation (data not shown). Further evidence that *Sp*Dre4-TAP associates with splicing complexes was the presence of U2, U5 and U6 snRNAs in the TAP eluate, as was found in the *Sp*Prp17-TAP, *Sp*Prp19-TAP, and *Sp*Cwf2-TAP eluates (Figure 5B). These associations have been conserved throughout evolution as a *Sc*Urn1-TAP eluate, contained a similar set of U2, U5 and NTC splicing factors as determined by 2D-LC-mass spectrometry (Figure 6 and Tables S4 and S5).

*Sc*Urn1 was previously identified in the inactive, but not the active, form of *S. cerevisiae* spliceosomal complex B whereas *Sc*Prp17 and *Sc*Cwc2 were identified in the active, but not inactive, complex B [5]. Similarly, we did not detect either *Sc*Prp17 or *Sc*Cwc2 in *Sc*Urn1-TAP complexes by mass spectrometric analysis (Figure 6). These results suggest that *Sc*Urn1 is not present in the same Prp19 complex as *Sc*Prp17 and *Sc*Cwc2. However, we did detect a low level of *Sp*Cwf2 in *Sp*Dre4-TAP complexes (Figure 3). Therefore, we tested whether *Sp*Dre4 could co-exist with *Sp*Prp17 and *Sp*Cwf2 in the same Prp19 complex. *S. pombe* Prp19-TAP complexes were isolated from *prp19-TAP cwf2-Myc dre4-GFP* and *prp19-TAP prp17-Myc dre4-GFP* cells. From these TAP eluates, *Sp*Dre4-GFP was able to co-immunoprecipitate *Sp*Cwf2 (Figure 5C) and *Sp*Prp17 (Figure 5D). From a *Sp*Prp19-TAP eluate, *Sp*Dre4 was also able to co-immunoprecitate *Sp*Cdc5, another *Sp*NTC component (Figure 5E). We were not able to detect these interactions in traditional co-immunoprecipitations, likely due to their low abundance and/or transient nature (data not shown). In combination, these results are consistent with the idea that Prp19 can associate simultaneously with all three of its identified WD40 binding partners at some stage in spliceosome assembly.

### *S. pombe* Saf1 and *S. cerevisiae* Aim4 functionally intersect with pre-mRNA processing

**Figure 7:**
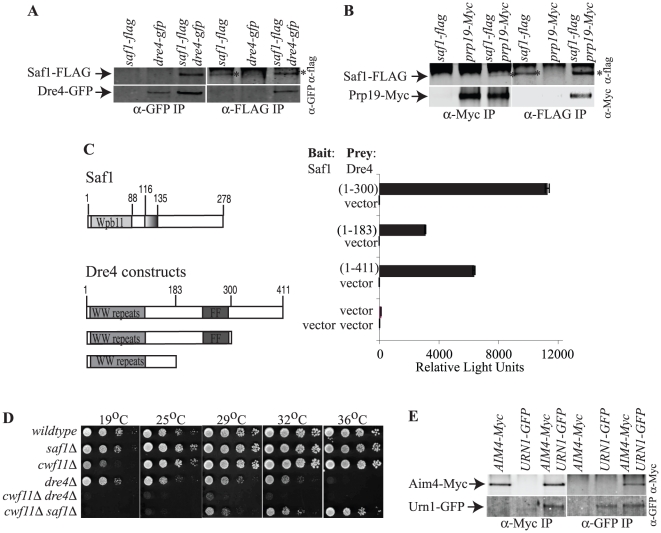
Characterization of *Sp*Saf1 and *Sc*Aim4. A and B) Anti-GFP (A) or anti-Myc (B) (left panels) or anti-FLAG (right panels) immunoprecipitates from the indicated *S. pombe* strains were blotted with antibodies to the FLAG epitope (top panels) or GFP (bottom panels). Asterisks mark the bands corresponding to *Sp*Saf1-FLAG. The band above it is the IgG heavy chain. C) *S. cerevisiae* strain pJ69-4A was cotransformed with bait plasmids expressing *Sp*Saf1 or nothing, and empty prey plasmid or prey plasmid expressing the indicated regions of *Sp*Dre4. ß-galactosidase activity (represented by relative light units) of the transformants is plotted. D) Equivalent cell numbers of the indicated *S. pombe* strains were spotted in 10-fold serial dilutions and incubated at the indicated temperatures for 3–5 days. E) Anti-Myc (left panels) or anti-GFP (right panels) immunoprecipitates from the indicated strains were blotted with antibodies to the Myc epitope (top panels) or GFP (bottom panels).

The protein identified in the *Sp*Dre4-TAP with the highest number of spectral counts and sequence coverage is an uncharacterized protein with a predicted molecular mass of 32 kDa and it was not identified in TAP complexes of *Sp*Prp19, *Sp*Cdc5, or *Sp*Prp17 (Figure 3, Tables S2 and S3). This ORF, SPCC663.11, was named Saf1 for Splicing Associated Factor 1. To confirm that *Sp*Saf1 was a *bonafide* partner of *Sp*Dre4, the *saf1^+^* open reading frame was tagged at its endogenous locus with three copies of the FLAG epitope and this allele was combined with *Sp*Dre4-GFP. In an anti-FLAG immunoprecipitate from the double tag strain, but not the single tag strains, both proteins were detected, and reciprocally, both *Sp*Saf1-FLAG and *Sp*Dre4-GFP were detected in an anti-GFP immunoprecipitate (Figure 7A). Thus, *Sp*Saf1 associates with *Sp*Dre4 and, as determined by directed two-hybrid analysis, the WW domain at *Sp*Dre4's N-terminus likely mediates *Sp*Saf1 interaction (Figure 7C). *Sp*Saf1 is a nuclear protein like *Sp*Dre4 (Figure S2D), and an interaction with *Sp*Prp19 can be detected as determined by co-immunoprecipitation (Figure 7B). The *saf1^+^* gene was deleted and found to be non-essential (Figure S2B). However, *saf1Δ* is synthetically lethal with *dre4Δ* and shows negative genetic interaction with the splicing mutant, *cwf11Δ* (Figure 7D). To determine the range of proteins associated with *Sp*Saf1, we expressed TAP-Saf1 in *saf1Δ* cells and purified TAP complexes. *saf1+* tagged at its endogenous C-terminus with the TAP epitope was not fully functional (data not shown). Several pre-mRNA splicing factors including *Sp*Prp19 were identified in TAP-Saf1 complexes by mass spectrometric analysis along with a number of background proteins typical of low abundance TAP eluates (Figure 3 and Table S3). These results are all indications that *Sp*Saf1 participates in some step of pre-mRNA processing.

To determine if a Saf1-like protein associates with *Sc*Urn1, we isolated *Sc*Urn1-TAP complexes and analyzed them by 2D-LC-mass spectrometry. The protein identified in the *Sc*Urn1-TAP with the highest number of spectral counts and sequence coverage was Aim4 (Figure 6 and Tables S4 and S5), a non-essential protein of unknown function. To confirm that Aim4 was a *bonafide* interacting partner of *Sc*Urn1, the *AIM4* open reading frame was tagged at its endogenous locus with the Myc_13_ epitope and this allele was combined with Urn1-GFP. In an anti-Myc immunoprecipitate from the double tag strain, but not the single tag strains, both proteins were detected, and reciprocally, both Aim4-Myc and Urn1-GFP were detected in the anti-GFP tagged immunoprecipitates (Figure 7E). Thus, *S. cerevisiae* Aim4 associates with Urn1 as does *S. pombe* Saf1 with Dre4. Purifications of Aim4-TAP contained a number of pre-mRNA splicing factors including Urn1 (Figure 6 and Tables S4 and S5) suggesting that it too connects to pre-mRNA splicing.

### Identification of *S. pombe* Saf2 and Saf3 as essential pre-mRNA splicing factors

**Figure 8:**
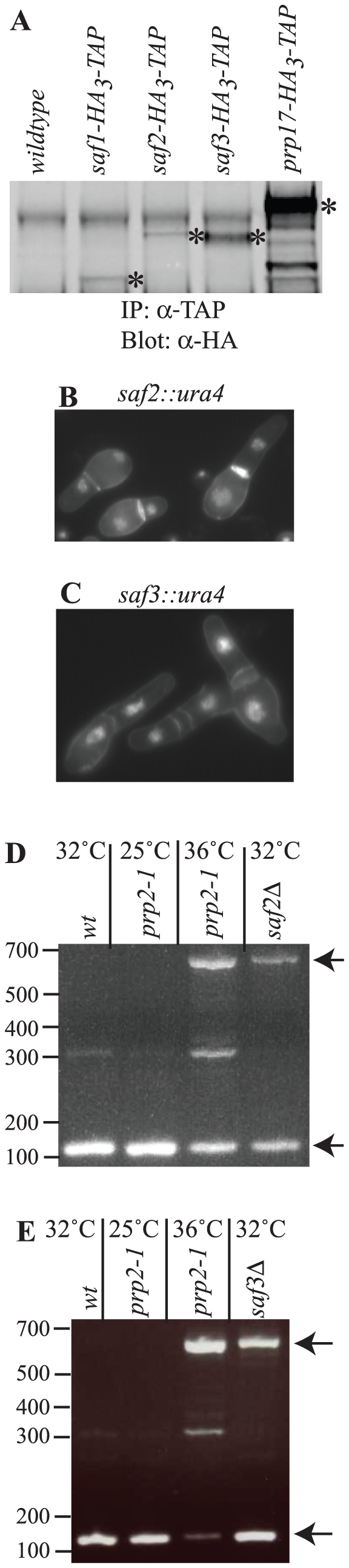
Characterization of *S. pombe* Safs. A) Protein G pull-downs from the indicated *S. pombe* strains were blotted with antibodies to the HA epitope. The bands with asterisks correspond to the indicated proteins and were quantified relative to background. B and C) Spores from the (B) *saf2::ura4^+^/saf2^+^* and (D) *saf3::ura4^+^/saf3^+^* diploids were germinated in minimal medium lacking uracil. Cells were fixed in formaldehyde at 15 and 40 h, respectively, and stained with DAPI. D and E) RNA was purified from wildtype cells grown at 32°C, *prp2-1* cells grown at 25°C(−) or shifted to 36°C (+) for 4 hours, or from spores germinated at 32°C for 24 h from *saf2::ura4^+^/saf2^+^* (D) and *saf3::ura4^+^/saf3^+^* (E) diploids in medium lacking uracil. RT-PCR reactions were performed using oligonucleotides that flank the long intron within the *prp19* mRNA. PCR products were separated on 3% Nusieve gels and detected with ethidium-bromide. Arrows indicate the position of prescursor and mature RNA species.

Two other predicted proteins were identified in the *S. pombe* NTC component TAPs discussed above with high sequence coverage (Figure 3 and Tables S2 and S3). Encoded by ORFs SPAC2F3.14c and SPAC1782.03, they have been called *Sp*Saf2 and *Sp*Saf3, respectively. To confirm that they interacted with *Sp*NTC components, they were tagged at their endogenous loci with the GFP, TAP, or HA_3_-TAP epitopes. Standard co-immunoprecipitations validated their interactions with *Sp*Dre4 and *Sp*Prp19 (Figure S3). Furthermore, following TAP, 2D-LC mass spectrometric analysis revealed that *Sp*Saf2 and *Sp*Saf3 associate with many splicing factors (Figure 3 and Table S3). There were also many background proteins identified, typical of low abundance proteins (Table S3). Indeed *Sp*Saf1, *Sp*Saf2 and *Sp*Saf3 are considerably less abundant proteins (43-, 55-, and 10-fold, respectively) than *Sp*Prp17 as determined by quantitative immunoblotting (Figure 8A). This prevented the determination of *Sp*Saf1, *Sp*Saf2 or *Sp*Saf3 TAP complex size by sucrose gradient sedimentation and clearly indicate that, like *Sp*Dre4, these proteins are not core NTC components.

To determine whether *saf2^+^* and *saf3^+^* played roles in pre-mRNA splicing, the genes were deleted from the genome. Both were found to be essential for viability. Spores lacking either *saf2^+^* or *saf3^+^* germinated and grew however typically arresting in the first cell cycle (Figure 8B and 8C). RT-PCR analyses of RNA isolated from these germinated spores demonstrated that both *saf2^+^* and *saf3^+^* are required for pre-mRNA splicing (Figure 8D and 8E).

## Discussion

Prp19 is the founding member of the NTC, and plays a central role in defining NTC architecture and function. The NTC is a group of proteins critical for the initiation of pre-mRNA splicing but the exact composition and function of the NTC remain unclear. Here, we combined a genome-wide two-hybrid approach with comparative proteomics of two evolutionarily distant yeasts to identify Prp19 binding partners. *Sc*Cwc2/*Sp*Cwf2, Prp17, and *Sc*Urn1/*Sp*Dre4 - all pre-mRNA splicing factors - were found to directly interact with the WD40 repeats of Prp19. In addition, three other *S. pombe* pre-mRNA splicing factors, Saf1, Saf2, and Saf3, that are broadly conserved and physically linked to the NTC, have been discovered by iterative proteomics. This work significantly augments our understanding of Prp19 connectivity and dynamic interactions within the spliceosome, and highlights the role of Prp19 as a central organizer of the NTC.

### Implications for NTC organization

Prp19 exists as an antiparallel tetramer with four independent WD40 domains, two at each end of the tetramer [11]. In a simple model, each Prp19 WD40 repeat would interact with one target, meaning that four copies of each WD40 binding protein could simultaneously bind to the Prp19 tetramer. Unexpectedly, however, both this and previous work indicate that the WD40 binding partners interact with Prp19 in distinctive ways. *Sc*Cwc2/*Sp*Cwf2 is present in a 1∶2 stoichiometry with tetrameric Prp19 [16], while we have shown here that at least four copies of Prp17 but only one copy of Urn1 interact with each Prp19 tetramer. In addition, our analysis of Prp19 complexes shows that NTC composition likely changes during different stages of spliceosome assembly with *Sc*Urn1/*Sp*Dre4, *Sp*Saf1, *Sp*Saf2, and *Sp*Saf3 interacting with Prp19 prior to spliceosome activation and other NTC components, such as *Sc*Cwc2/*Sp*Cwf2 and Prp17 associating with Prp19 later. The ability of the Prp19 WD40 repeats to dynamically interact with a number of binding partners, each with distinct stoichiometries and at distinct stages of pre-mRNA splicing, may be a mechanism for coupling structural rearrangements within the NTC directly to its function during the splicing reaction. Although we did not detect the orthologous interaction in either *S. pombe* or *S. cerevisiae*, human Prp3 is reported to interact with the human Prp19 WD40 domain suggesting even additional complexity in modulating Prp19 function through WD40 binding partners [15].

**Figure 9:**
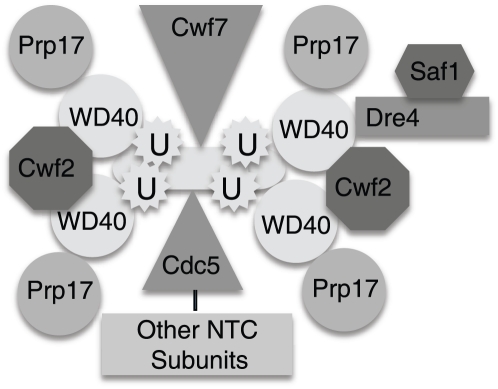
Model of Prp19 organization in the *S. pombe* NTC.

Conserved from yeasts to human [59], [60], [61], Prp17 is considered to be a second step factor present only in activated spliceosomes (for example, [5]) although other evidence suggests it associates with snRNPs prior to the first step of splicing [48], [52]. Here, we establish that Prp17 binds directly to the Prp19 WD40 domain and is present in splicing complexes in a 1∶1 stoichiometry with Prp19. Based on the combined evidence, we conclude that Prp17 is a *bona fide* NTC component, constitutively associated with Prp19 (Figure 9). The Prp17 N-terminus, which interacts with Prp19, does not encode a recognizable motif but some temperature-sensitive *prp17* mutations map to this region [62]. Given the Prp17 domain architecture, and numerous genetic interactions between *prp17Δ* and other splicing mutants [62], [63], it is likely that Prp17 stabilizes associations among NTC components or between the NTC and snRNPs. It will be interesting to determine if Prp17 associates with other splicing factors through its WD40 domains and if so, to learn their identities.

### Characteristics of new pre-mRNA processing factors

A mutation in *S. pombe dre4^+^*, *dre4-54*, was isolated in a screen for DNA replication factors but *dre4-54* cells display heterogeneous phenotypes [56]. Such varied phenotypes can arise from defective pre-mRNA splicing [64] if spliceosome assembly is affected. The predicted consequence of defective spliceosome assembly is that variable mRNAs become limiting at different times, and the cells eventually die from accumulated defects in multiple processes. As measured by accumulation of pre-mRNAs, the defect in pre-mRNA splicing in *dre4* mutants is modest. However, because erroneously processed pre-mRNAs can be expeditiously degraded by the exosome (reviewed in [65]) and/or the nonsense mediated mRNA decay pathway [66], an important role for Dre4 in pre-mRNA splicing is not ruled out by this observation. Indeed, based on associated proteins and synthetic sick interactions between *urn1Δ* and mutations in *S. cerevisiae its3, isy1, pml1* and *snu66* [58], and *dre4Δ* with *S. pombe* splicing mutants shown here, the most parsimonious explanation for *Sc*Urn1/*Sp*Dre4 function is as a splicing factor. We have narrowed *S. pombe* Dre4's important functional region to that containing the FF and WW domains, which suggest it is likely to promote protein-protein interactions within splicing complexes.

Possible *Sp*Saf1 homologs sharing a WW binding protein 11 domain exist in a variety of eukaryotes (Figure S4A), but not *S. cerevisiae*. A human relative variously called SNP70/SIPP1/NpwBP/Wbp11 has been implicated in pre-mRNA splicing [67], [68], [69] and was identified in purifications of the human spliceosome [70], [71]. Although *S. cerevisiae* Aim4 does not contain a WW binding protein 11 domain, it might be the *S. cerevisiae* Saf1 homolog. We base this prediction on its significant co-purification with *Sc*Urn1, its identification as a *Sc*Urn1-interacting protein in global association studies [26], [46], its interaction with many pre-mRNA splicing factors, and its sequence similarity with *Sp*Saf1 (Figure S4B). Taking this evidence into account, it seems likely that *S. pombe* Saf1 and *S. cerevisiae* Aim4 might affect pre-mRNA processing through their interaction with *Sp*Dre4 and *Sc*Urn1, respectively.

*Sp*Saf2, which contains predicted coiled-coil and WW domains, is conserved in other fungi (Figure S5) but we did not detect sequence homologs in higher eukaryotes or *S. cerevisiae*. We speculate, however, that a functional ortholog might well exist based on *Sp*Saf2's essential function, and further comparative proteomic analyses might reveal its identity.

*Sp*Saf3 is a highly conserved protein (Figure S6) although an obvious analog cannot be identified in *S. cerevisiae*. The Saf3 human ortholog was mistakenly implicated in Marfan syndrome [72] and named microfibrillin-associated protein-1 (MFAP1) [73]. However, *Drosophila* MFAP1 is essential for pre-mRNA splicing [74] and hMFAP1 was identified by mass spectrometry in numerous purifications of the human spliceosome [70], [74], [75], [76], [77]. *Drosophila* MFAP1 binds directly to Prp38 [74] and this relationship is likely to be conserved based on the good recovery of *Sp*Prp38 and *Sp*Snu23 in the *Sp*Saf3-TAP (Figure 3 and Table S3). It will be interesting to determine the exact connectivity between *Sp*Saf3 and the *Sp*NTC in future studies.

A notable feature of Dre4, Saf1, Saf2, and Saf3 is their low abundance relative to other *S. pombe* NTC splicing factors suggesting that they are not stoichiometric NTC components. Their low abundance is also a reasonable explanation for why they were not detected by mass spectrometry in TAP eluates of NTC components [24] (Figure 3). Based on the compilation of co-purifying proteins, we infer that Saf1, Saf2 and Saf3 interact with the *S. pombe* spliceosome early in its assembly and Dre4 before its activation. Indeed, *Sc*Urn1 was recently identified in *S. cerevisiae* complex B but not in activated forms of the spliceosome whereas *Sc*Prp17 and *Sc*Cwc2 are reported to be present in *S. cerevisiae* spliceosomes only after their activation [5]. Our findings in concert with others suggest that understanding the mechanisms governing Prp19 WD40 binding to Prp17, *Sc*Cwc2/*Sp*Cwf2, and *Sc*Urn1/*Sp*Dre4 might be important for fully understanding spliceosome activation.

## Supporting Information

Figure S1 **Sequence alignment of** ***Sp*** **Dre4 and** ***Sc*** **Urn1.** Identical residues are indicated in red.
(EPS)

Figure S2 **Characterization of Prp19 associated proteins.** A) *S. cerevisiae* strain pJ69-4A was cotransformed with bait plasmids expressing the indicated regions *Sc*PRP17 with prey plasmid expressing full-length *Sc*PRP19. + indicates growth and – denotes no growth on selective media. ß-galactosidase activity (represented by relative light units) of the transformants is plotted in the right panels. The line indicates the region of Prp17 sufficient for interaction in this assay. B) The indicated strains were struck to YE agar plates and incubated at either 25°C or 36°C. C) *dre4Δ* cells were transformed with the indicated vectors and transformations were streaked and incubated at the indicated temperatures. D) Live cell images of the indicated strains.
(EPS)

Figure S3 ***S. pombe*** **Safs interact with Dre4 and Prp19.** A) Anti-Myc (left panels) or anti-GFP (right panels) immunoprecipitates from the indicated strains were blotted with antibodies to the Myc epitope (top panels) or GFP (bottom panels). B and D) Anti-Myc (left panels) or anti-GFP (right panels) immunoprecipitates from the indicated strains were blotted with antibodies to the Myc epitope. C) Anti-Myc (left panels) or anti-TAP (right panels) immunoprecipitates from the indicated strains were blotted with IgG that recognizes the TAP epitope.
(EPS)

Figure S4 **Sequence alignment of Saf1 homologs.** A) MultAlin-generated sequence alignment of *S. pombe* (S. pom), *Schizosaccharomyces japonicus* (S. jap) yFS275, *Aspergillus nidulans* (A. nid) AN8724.2, *Ustilago maydis* (U. may) UM03371.1, and human (Wbp11) Saf1 homologs. Residues with high sequence identity or conservation are in red and those with lower sequence identity are in blue. B) A region of sequence similarity in Saf1 is shared with Aim4 and the other indicated proteins.
(DOC)

Figure S5 **Sequence alignment of Saf2 homologs.** MultAlin-generated sequence alignment of *S. japonicus* (S. jap), *S. pombe* (S. pom), *Schizosaccharomyces octosporus* (S. oct), *A. nidulans* (A. nid) AN8804.2, *Talaromyces stipitatus* (T. sti) XP_002482024.1, and *Coccidioides immitis* (C.imm) XP_001242717.1 Saf2 homologs. Residues with high sequence identity or conservation are in red and those with lower sequence identity are in blue.
(DOC)

Figure S6 **Sequence alignment of Saf3 homologs.** MultAlin-generated sequence alignment of Saf3 homologs from *S. pombe* (S. pom), human NP_005917.2, *Xenopus laevis* (frog) NP_001080142.1, *Drosophila melanogaster* (fly) NP_647679.1, and *Caenorhabditis elegans* (worm) F43G9.10. Residues with high sequence identity or conservation are in red and those with lower sequence identity are in blue.
(DOC)

Methods S1 Supplementary Methods
(DOC)

Table S1 **Yeast strains used in this study.**
(DOC)

Table S2 **Heatmap of 100 most abundant proteins identified from Cwf2-, Prp17-, and Prp19- TAPs.** “ORF” = open reading frame, “% Coverage” = % sequence coverage from MS analysis, “TSC” = total spectral counts, and shaded cells indicate protein abundance index (PAI, spectral counts/distinct peptides) numbers (Ref. 71) for the TAPs indicated at the top of each column.
(PDF)

Table S3 Heatmap of splicing-associated proteins identified from Dre4-, Saf1-, Saf2-, and Saf3- TAPs. “ORF” = open reading frame, “% Coverage” = % sequence coverage from MS analysis, “TSC” = total spectral counts, and shaded cells indicate protein abundance index (PAI, spectral counts/distinct peptides) numbers (Ref. 71) for the TAPs indicated at the top of each column.
(PDF)

Table S4 Splicing-related proteins identified from Aim4- and Urn1- TAPs. “ORF” = open reading frame, “% Coverage” = % sequence coverage from MS analysis, “TSC” = total spectral counts, and shaded cells indicate protein abundance index (PAI, spectral counts/distinct peptides) numbers (Ref. 71) for the TAPs indicated at the top of each column.
(PDF)

Table S5 **Heatmap of other (non-splicing) proteins identified from Aim4- and Urn1- TAPs.** “ORF” = open reading frame, “% Coverage” = % sequence coverage from MS analysis, “TSC” = total spectral counts, and shaded cells indicate protein abundance index (PAI, spectral counts/distinct peptides) numbers (Ref. 71) for the TAPs indicated at the top of each column.
(PDF)
