# Correction: Systematic Two-Hybrid and Comparative Proteomic Analyses Reveal Novel Yeast Pre-mRNA Splicing Factors Connected to Prp19

**DOI:** 10.1371/annotation/62e74a1c-c922-495b-9aaf-eebfe6b85ff5

**Published:** 2011-09-02

**Authors:** Liping Ren, Janel R. McLean, Tony R. Hazbun, Stanley Fields, Craig Vander Kooi, Melanie D. Ohi, Kathleen L. Gould

There are multiple errors in the affiliations. The correct affiliation for Liping Ren, Janel R. McLean, and Kathleen L. Gould is: Howard Hughes Medical Institute, Vanderbilt University, Nashville, Tennessee, United States of America. The correct affiliation for Tony R. Hazbun and Stanley Fields is: Howard Hughes Medical Institute, University of Washington, Seattle, Washington, United States of America 

