# Supplementary material for: Systematic Two-Hybrid and Comparative Proteomic Analyses Reveal Novel Yeast Pre-mRNA Splicing Factors Connected to Prp19
Source: PLoS One. 2011 Feb 28;6(2):e16719. doi: 10.1371/journal.pone.0016719 (PMC3046128; doi:10.1371/journal.pone.0016719)
Supplement: Figure S4 — Sequence alignment of Saf1 homologs. A) MultAlin-generated sequence alignment of S. pombe (S. pom), Schizosaccharomyces japonicus (S. jap) yFS275, Aspergillus nidulans (A. nid) AN8724.2, Ustilago maydis (U. may) UM03371.1, and human (Wbp11) Saf1 homologs. Residues with high sequence identity or conservation are in red and those with lower sequence identity are in blue. B) A region of sequence similarity in Saf1 is shared with Aim4 and the other indicated proteins. (DOC) [file pone.0016719.s004.doc]

A S. pom ML SKRGLNPVQA ESRKKKKREQ IKVREERAKR HEERLSHRNM SQMERQLSEL TQLESS-QAL NAHDKQLLQN -71

S. jap MK GKRSLNPVQD EARKRKKKEQ AKLRQERSQR RDAKLSTKNV FFLEKKIFEL KDIEKS-RPL NSSERQQLDT -71

A. nid MAKD KERSINPAAA QRKLEKQKSL KKSKAEALAR RNEKLARRNP ERIQRQINEL KEMEQSGQSL RPREKQILEA -74

U. may MVKSTDPVQA ARRAAIKREA NRNRERRQQA NDARALRQDT SSLERQIQRL QSRGAALDQQ DQEELKRLQD -70

Human MGRRSTSSTK SGKFMNPTDQ ARKEARKREL KKNKKQRMMV RAAVLKMKDP KQIIRDMEKL DEMEFNPVQQ PQLNEKVLKD -80

S. pom LQRDM-AIMK KKNIHGHRVG RVESDKTKEA ERQHKPRKPF I------PKN PKRSIYYDPI FNPYGVPPPG MPYREKEE-- -142

S. jap LERDV-YVMK KKGIGGHSIG NIESEKTKHV NRERRQKKPF I------PKN PKKSVYYHPI FNPYGVPPPG MPYKEIDDDN -144

A. nid LERDLRAVLK AREALGDKAP KFASSQHQRG DDHPRERRDG G------VLG KRRRDNHG-- ---------- ----RFGDQD -128

U. may EVANVN---- RIKEEYIRKH PDQRNFVRGY EEPSSNSDNQ I------VTT STSIAVRNTT R-NVPAAASQ TTSRD-PRWS -138

Human KRKKLRETFE RILRLYEKEN PDIYKELRKL EVEYEQKRAQ LSQYFDAVKN AQHVEVESIP LPDMPHAPSN ILIQDIPLPG -160

S. pom -LSSETDESV IDIPLPSEEY PFEDPKPREK KNKSFKPKHH KKQDINASSA QPKSTTTTEA AANTKDIEEE TMIEYSAQPV -221

S. jap SESSETDESV INIPMPEDEF PGDNPHHEDT KRTAL----- ----VEHNDV WKQQEITADA IVKT------ ---EYSAEPI -206

A. nid SDSSETDEEV RRIPMPRDTP PPIPREYQRR KEANAGGQAR GPHPLPAKPL VTESKTVYEA KPEIRDLRKE AVSKFI--PA -206

U. may IYYDAVFNPY GA-PPPGMPY LEKPHAQLVQ EGLLDAAKPP -PLPEETEAQ GLSLDDDSDD SSIDEDLK-- -DIIMPSGPP -213

Human AQPPSILKKT SAYGPPTRAV SILPLLGHGV PRLPPGRKPP GPPPGPPPPQ VVQMYGRKVG FALDLPPRRR DEDMLYSPEL -240

S. pom VRDLRQEAAQ FLPAAFQRQK LAKGQKIGQP DRDVSSQVQE DKDEEIDNFY KEIGGYL -278

S. jap VRDLRKEAAQ FIPASLKRQP ----QQLEEP NADSELEKYY SELNNLGASD SNPSHDK -259

A. nid AVRVKKESIR GQGKLLEPEE LDRLEKAGYN AGPAETTTTA DEQSRLLEEE RRFDQELKSV QIEEVEDEEA -276

U. may PIRLLTETMP SSRPSTLLRS ARGQGRGRGR GQAAMERRGF GSSRGSREPG SGSLHQANGG RQHAGFDQRA . . . . . -442

Human AQRGHDDDVS STSEDDGYPE DMDQDKHDDS TDDSDTDKSD GESDGDEFVH RDNGERDNNE EKKSGLSVR- F . . . . -561

B S. cerevisiae Aim4 P38305.1/71-94     ELGIRSIFYDKDWNPRGTAPSHYR...
 C. albicans C4YID5.1/106-129   LWGQKSIYFNPELNPLGKVPE---len
 K. thermotolerans C5DII3.1/30-53     ELGERSIFYDHEWNPEGKAPSGYK...
 K. polyspora A7TGL9.1/42-65     ELFRKSIFFDSHWNPNGVAPPNHR...
 P. stipitis A3LU98.2/106-129   LHGSKSIYFNPELNPLGKIPNPD-a..
 K. lactis B4UN83.1/38-61     ELGIKSIFYDPDWNPSGKAPFGLK...
 C. albicans Q59TD2.1/106-129   LWGQKSIYFNPELNPLGKVPE---len
 C. glabrata Q6FJL9.1/78-101    ELGPKSIFYDPDWNPKGEAPDGFR...
 D. hansenii B5RUC4.1/106-127   LWGSKSIYFNPELNPLGKVP----ge.
 S. cerevisiae A6ZLD4.1/71-94     ELGIRSIFYDKDWNPRGTAPSHYR...
 C. tropicalis C5M6W9.1/106-129   LWGSKSVYFNPELNPLGKVPVI--nn.
 P. guilliermondii A5DDX4.1/103-126   LWGMKSVYFNPELNPLGKVPQISQ...
 Z. rouxii C5DP04.1/9-32      ELGSKSIFYDPEWNPKGLAPPGFK...
 S. cerevisiae B3LMW8.1/71-94     ELGIRSIFYDKDWNPRGTAPSHYR...
 C. lusitaniae C4YAF3.1/133-156   LWGKESVYFNPELNPLGKVPDWRN...
 S. pombe Saf1 O74517.1/116-135   ---KRSIYYDPIFNPYGVPPPG--m..
 P. pastoris C4R4M4.1/10-33     ALGRRSIFYEPQWNPSGKAPKGFK...
 C. dubliniensis B9WBC2.1/106-129   LWGQKSIYFNPELNPLGKVPQ---vgn

Figure S4
