# Supplementary material for: Systematic Two-Hybrid and Comparative Proteomic Analyses Reveal Novel Yeast Pre-mRNA Splicing Factors Connected to Prp19
Source: PLoS One. 2011 Feb 28;6(2):e16719. doi: 10.1371/journal.pone.0016719 (PMC3046128; doi:10.1371/journal.pone.0016719)
Supplement: Figure S5 — Sequence alignment of Saf2 homologs. MultAlin-generated sequence alignment of S. japonicus (S. jap), S. pombe (S. pom), Schizosaccharomyces octosporus (S. oct), A. nidulans (A. nid) AN8804.2, Talaromyces stipitatus (T. sti) XP_002482024.1, and Coccidioides immitis (C.imm) XP_001242717.1 Saf2 homologs. Residues with high sequence identity or conservation are in red and those with lower sequence identity are in blue. (DOC) [file pone.0016719.s005.doc]

S.jap MNNGAE TAPEITYAVI KPANADGFMI NERAMEQTNN NSPRSERGYN SELAKKPTK- -55

S.pom MSSSK DCKATSNVDQ TIPASNVNSG DFISSNTSSS NSENSNIQGK HYTQVGEDAD NSFISENTPK NTFESTQTYE -75

S.oct MEQEKNISTL ECKSTDLCIS SRPSKESSPN PIEPLGKQDT PHSPSQKRTE H---IGESGS QPPLPDEPIP EEYQTKST-- -75

A.nid MSTSHTN DAPEGASGSA SPSPDTTSPS R----DNDPT VTTKAEKQAE EHTSEQKSE- -52

T.sti MAEN DRLAVADTTE KSSTSASNTD QPGDDSSRTE HREEVEAEES DGVETTKQRR PEAGEEKGE- -63

C.imm MS SPDPEEGQPS NVHKPEMEED RPAANEKSKG GETGQQET-- -40

S.jap --NESSFAPP LPDEPVPKLE QSQANQ---S ETELLKNGTG IVLPVPKMNG RMNSPSFSEE ST-------L NNADLSAPPL -123

S.pom NLESISKNEP TSEASKPLLN ELVPEEPLPR EPPLPNEPVP EE--PLPGEP PLPDEPVPEE PLPGEPPLPN EPVPETNCH- -152

S.oct --EGPESQPP LPDEPLPEEY QGPAHKEDDA QPPLPDEPIP EEYQTKSTEG PESQPPLPDE PLPEEYQGPA HKEDDAQPPL -153

A.nid --DSTPQQEE GEQEEGETRE SDAPPLPDEV PPPLPNEAPP GE-----DDG WEPVWDANAQ AYY------F YNRYTGVSQW -119

T.sti --DGSEEKRE VE-ETGEVNT EEAPPLPDEE VPPLPDEAPP GQEEQQGDDG WEPVWDANAQ AYY------F YNRITGVSQW -134

C.imm --DGKKSGED TQDGDAETRV SAPPLPAEEA PPPLPNEPIP GGAE---DDG WDALWDDSAQ AYY------F YNRFTGVSQW -109

S.jap PDEPIPQTEA ETQPPLPDEP VPVSESTEKP RMAWSADGQV AAIWDDATEA YYFWDKRTNT TSWENPLD-- EKQEEDQEDY -210

S.pom KESPLSDETV SETSKNDTSN SPTNENQAQP SIAWSEGHRI AAIWDPSQQA YYFWDTLTNT TSWNNPLED- EEQTSPLD-Y -230

S.oct PDEPIPEKSK SIDNDTDPSI SGADVLSSQP SIAWSEDQQV AAIWDNNAQA YYFWDRRTNT TSWENPLETA QSEEEPSDTY -233

A.nid ENPRVPDAAV ATAAAPPAV- GTEEPAPAEK ASAPLGGYNP AIHGDYDPTA PY---AQQYE RQEEGIHGGA GMGLVNTAGY -195

T.sti ENPRVPDAA- -SVSAPRAQ- KVEEQVIR-- -----GGYNP AIHGDYDPTA PY---AQQYE ADLQ--LASA VNAADQSQSY -199

C.imm ENPRIPDAQQ GPPGVESEKD GAEETQQQRP RPRVAGGYDP AIHGDYDPDA WY---AQPHP DD------TS PASTDPSVLY -180

S.jap TSVVRLSKLT GKFIRPEATP ETQSEPQKAY KHMEQFFDVK GHLQEHNGKS LLEERRNKRY TRKEMAELKR KAKERKERKR -281

S.pom TAKVQFNRLS GKFMPKWASP ELRSEENKAH KHMEQYFDIN SSLNSHNGQS LLAERRNKRY TRKEMEQMKR RTKEKKEMKR -310

S.oct ATRVQFNRLS GKYMPDWATP EMKSEEYKAH KHMGQFFDVD AAMSSTGGKS LLEERRNKKY TRKEMEQMKK RNKEKKEQKR -313

A.nid EAIGSFNRFT GRWQAASLNP EYHNDENKSR RQMNAYFDVD AAANAHDGRS LRAERSAKKL SKKELKMFKD KRREKKEEKR -275

T.sti TATGAFNRFT GRWQDSSLTP ENFNDENKSR RQMNAYFDVD AAANSHNGRS LKAERSHKKL TKAEVKAFRE KRRERKEEKR -279

C.imm TATGAFNRFT GKWQPLGLTP ENFNDENKSR RQLNAYFDVD AAANSHEGRS LKAERSGKKL TKKELKAFKE KRREKKEEKR -260

S.jap RALFDISSDD IDFRRRKIIR Y -302

S.pom RALYDIASDE KDFRRRKIIR Y -331

S.oct RAMFDIASDD KDFRRRKIIR Y -334

A.nid RAWLRD -281

T.sti RAWLRD -285

C.imm RAWLRD -266

Figure S5
