# Supplementary material for: Systematic Two-Hybrid and Comparative Proteomic Analyses Reveal Novel Yeast Pre-mRNA Splicing Factors Connected to Prp19
Source: PLoS One. 2011 Feb 28;6(2):e16719. doi: 10.1371/journal.pone.0016719 (PMC3046128; doi:10.1371/journal.pone.0016719)
Supplement: Figure S6 — Sequence alignment of Saf3 homologs. MultAlin-generated sequence alignment of Saf3 homologs from S. pombe (S. pom), human NP_005917.2, Xenopus laevis (frog) NP_001080142.1, Drosophila melanogaster (fly) NP_647679.1, and Caenorhabditis elegans (worm) F43G9.10. Residues with high sequence identity or conservation are in red and those with lower sequence identity are in blue. (DOC) [file pone.0016719.s006.doc]

Human M SVPSALMKQP PIQSTAGAVP VRNEKGEISM EKVKVKRYVS GKRPDYAPME SSDEEDEEFQ FIKKAKEQE- -70

Frog MA SSANALNKLP PIQSTAGAVP VRNEKGEISM EKVKVKRYVS GKRPDYAPME SSDEEEEEFQ FIKKGKEQE- -71

Fly M SAATAAAAAS GIQSTAGAIP MRNEKGELSM QKVKVQRYIS GKRPDYARAD SSSEESDDDD FIDTRKRLER -71

Worm MGDYVPGFEQ RESDNRSFGH SRLPTLGAIP IKNEKGQTVM QKVKVSRYVA GKAPEYARNY DSDSSESDRE TDRDDDRRRR -80

S.pom MPIDT PLIPHKPISR YRSGRLEGLE SESSSESDYE -35

Human ---------- ---------- ----AEPEEQ EEDSSSDPRL RRLQNRI--S EDVE-ERLAR HRKIVEPEVV --GESDSEVE -121

Frog ---------- ---------- ----VEQEEQ PEESTSDPRL RRLQSRM--N EDVE-ERLAR HRKIIEPEVV --GESESEPE -122

Fly HKAERHKLEL SRQGGSAEGE ERAAGEGQEE DDAEVDDPRL RRLRQRPVDM EDMERERRER HRHIHEPEIM ESDSEDEEED -151

Worm RRRE------ ---------- --SSDEEDRR RHRRHEDYGR RRQVEKPEVL GKVEDESSEN EQESEEDE-- --EKQEERRE -138

S.pom EVSDSHDQEN SIS---SSRH VITPVFEKEG ISETKTSSNF QNINPVQTID NSASEYETDA SSAEGGSNSA ASSSEEEDSS -112

Human -GDAWRMERE D------SSE EEEEEIDDEE IERRRGMMRQ RAQERKNE-E MEVMEVEDEG RSGEESESES EYEEYTDSED -194

Frog PAEDWRMDRE V------TSE EEEEEVDDEE IEQRRSMMRQ RAAERKNE-E MEVMEVEDEG RSGEESASES EYEEYTDSED -198

Fly EGAQGAIQRG TNKITLASES DTDAELSDTE LENRRTKLRS RMLQQQRE-E -EVLQKEDEK QSESSESESS EYEEETESEE -229

Worm RARMRRLELH ENNREKDEEQ EDSAESDEED FERRRQMLRD RAIKREEEIK REIKEELEED DVEEEEEEES SEEEDSDEDD -218

S.pom DSEYEME--- -LRRRTLLLP PKFTSKVIKN RAKANEEDTE VLKKVTSQKI LEETIKRELL LKETKNNNEL LNDIDDTDGI -188

Human EMEPRLKPVF IRKKDRVTVQ EREAEALKQK ELEQEAKRMA EERRKYTLKI VEEETKKELE ENKRSL--AA –LDALNTDDE -271

Frog EMEPRLKPVF IRKKDRVTVQ EKEAEAIKQK ELDAEAKRMA EERRKYTLKI VEEETRKEIE ENKRSL--AA –LDALNTDDE -278

Fly DNEPRLKPLF VRKRDRATIQ EKEREAQKQK QLEAEAKRAA KERRRATLRM VEESVKKDLE KTKPETNEAC –IEDVCTDDE -308

Worm DPVPRLKPIF TRKKDRITLQ EAEKEKEKEI LKKIEDEKRA EERKRESAKL VEKVLQEEEA AEKRKTEDRV DLSSVLTDDE -298

S.pom DPQSEYELWK LRHLLRKKRD KEKSLELERE KMAIEERRLM NSEEREAQDL KDAEASRRGK KKSSMQFLQK YYHKGAFYQN -268

human NDEEEYEAWK VRELKRIKRD REDREALEKE KAEIERMRNL TEEERRAELR ANGKVITNKA VKGKYKFLQK YYHRGAFFMD -351

Frog NDEEEYEAWK VRELKRIKRD REEREAMEKE KAEVDRLRNM TDEERRAELR ANGKIITNKA MKGKYKFLQK YYHRGAFFLD -356

Fly NDEVEYEAWK LRELKRMKRD REERDNVERE KLDIDRMRNM TEEERRQELR QNPKVVTNKA TKGKYKFLQK YYHRGAFYLD -388

Worm TENMAYEAWK LREMKRLKRN RDEREEAARE KAELDKIHAM SEEERLKYLR LNPKVITNKQ DKGKYKFLQK YFHRGAFFLD -378

S.pom EDI-VSKRDY SEATEGEVLN KDLLPKPMQI RGDLFAKAGQ TRWTHLANED TTKEGSAWYD PKNPILQKNL HRLGGLHS-- -345

Human EDEEVYKRDF SAPTLEDHFN KTILPKVMQV KN--FGRSGR TKYTHLVDQD TTSFDSAWGQ ESAQNTKFFK QKAAGVRDVF -429

Frog EDENVYKRDF SAPTLEDHFN KTILPKVMQV KN--FGRSGR TKYTHLVDQD TTSFDSAWGQ DNPQNTKFFK QKAAGVRDVF -356

Fly EENDVLKRDF AQATLEDHFD KTILPKVMQV KN--FGRCGR TKYTHLVDQD TTKFDSPWYA ESSSNIKFHN EHAGGMRQQF -466

Worm EEDEVLKRNF AEATNDDQFD KTILPKVMQV KN--FGKASR TKYTHLTEED TTDHQGVWAS TNQLNSQFST KRAGGSRPVF -456

S.pom DSPLSKRKRT -355

Human ERPSAKKRKT T -440

Frog ERPSVQKRKT T -367

Fly DKPTGSKRKK ME -478

Worm ERPATKKRKN -466

Figure S6
