# Supplementary material for: Systematic Two-Hybrid and Comparative Proteomic Analyses Reveal Novel Yeast Pre-mRNA Splicing Factors Connected to Prp19
Source: PLoS One. 2011 Feb 28;6(2):e16719. doi: 10.1371/journal.pone.0016719 (PMC3046128; doi:10.1371/journal.pone.0016719)
Supplement: Methods S1 — Supplementary Methods (DOC) [file pone.0016719.s007.doc]

**Supplementary Methods**

**Microscopy Methods**. Cells in mid-log growth were imaged live at 25ºC using an Ultraview LCI spinning disk confocal microscope (Perkin Elmer) with a 100X/1.40 Plan-Apochromat oil immersion objective and a 488 nm argon ion laser to visualize GFP-tagged proteins.

**MS data analysis.** MS data were generated and prepared for processing as described in “Materials and Methods.” MS data analysis was performed as described in “Materials and Methods” with the following changes. Protein identification was performed with the Myrimatch algorithm (v1.6.33, [1]. Myrimatch parameters were as follows: strict tryptic cleavage; modification of methionine (oxidation, dynamic modification, +16 Da), Ser/Thr/Tyr (phosphorylation, dynamic modification, +80 Da) and cysteine (carboxamidomethylation, static modification, +57 Da) was allowed; precursor ions were required to be within 0.6 *m/z* of the peptide monoisotopic mass; fragment ions were required to fall within 0.5 *m/z* of the expected monoisotopic mass. IDPicker v2.6.114 [2,3] was used to filter peptide matches with the following parameters: max. FDR per result 0.02, max. ambiguous IDs per result 2, min. peptide length per result 5, min. distinct peptides per protein 3, min. additional peptides per protein group 2, min. number of spectra per protein 3, indistinct modifications M 15.994 Da, C 57.05 Da and distinct modifications S/T/Y 80 Da. Contaminant proteins (*e.g.* keratin, IgG) and nonspecific background proteins (identified by performing a TAP/LC-MS/MS in an untagged strain) were removed from the final protein identification lists. Protein abundance index (PAI = spectral counts/distinct peptides) numbers [4] were generated for each protein to normalize across datasets before generation of supplemental tables in Excel (Microsoft®, 2007).

**Other methods.** Sequence alignments were formulated at <http://bioinfo.genopole-toulouse.prd.fr/multalin/multalin.html> [5].

1. Tabb DL, Fernando CG, Chambers MC (2007) MyriMatch: highly accurate tandem mass spectral peptide identification by multivariate hypergeometric analysis. J Proteome Res 6: 654-661.

2. Zhang B, Chambers MC, Tabb DL (2007) Proteomic parsimony through bipartite graph analysis improves accuracy and transparency. J Proteome Res 6: 3549-3557.

3. Ma ZQ, Dasari S, Chambers MC, Litton MD, Sobecki SM, et al. (2009) IDPicker 2.0: Improved protein assembly with high discrimination peptide identification filtering. J Proteome Res 8: 3872-3881.

4. Rappsilber J, Ryder U, Lamond AI, Mann M (2002) Large-scale proteomic analysis of the human spliceosome. Genome Res 12: 1231-1245.

5. Corpet F (1988) Multiple sequence alignment with hierarchical clustering. Nucleic Acids Res 16: 10881-10890.
