# Supplementary material for: Systematic Two-Hybrid and Comparative Proteomic Analyses Reveal Novel Yeast Pre-mRNA Splicing Factors Connected to Prp19
Source: PLoS One. 2011 Feb 28;6(2):e16719. doi: 10.1371/journal.pone.0016719 (PMC3046128; doi:10.1371/journal.pone.0016719)
Supplement: Table S1 — Yeast strains used in this study. (DOC) [file pone.0016719.s008.doc]

**Table S1 Strains used in this study**

**Strain Genotype Source**

***S. pombe* strains**

KGY246 *ade6-M210 ura4-D18 leu1-32* h^-^ Lab stock

KGY2312 *prp19-myc_13_::kan ^R^ ade6-M21X ura4-D18 leu1-32* h^+^ Lab stock

KGY3369 *cwf11::ura4^+^* *ade6-M21X ura4-D18 leu1-32* h^-^ This study

KGY4244 *smg1-myc_13_::kan ^R^ ade6-M210 ura4-D18 leu1-32* h^-^ This study

KGY5687 *prp17-myc_13_::kan ^R^ ade6-M210 ura4-D18 leu1-32* h^+^ This study

KGY5693 *prp17-TAP::kan ^R^ ade6-M210 ura4-D18 leu1-32* h^+^ This study

KGY5850 *prp17-myc_13_::kan ^R^ prp19-myc_13_::kan ^R^ cwf7-HA_3_::Kan^R^* This study

*ade6-M21X ura4-D18 leu1-32* h^-^

KGY6440 *prp19-GFP::kan ^R^ prp17-myc_13_::kan ^R^* This study

*ade6-M21X ura4-D18 leu1-32* h^+^

KGY6464 *dre4-54 ura4-D18 ade6-M21X leu1-32 can1-1* h^-^S. Forsburg

KGY6467 *dre4-myc_13_::kan ^R^ ade6-M210 ura4-D18 leu1-32* h^+^ This study

KGY6468 *dre4-GFP::kan ^R^ ura4-D18 leu1-32* *ade6-M210* h^+^ This study

KGY6488 *dre4-GFP::kan ^R^ prp19-myc_13_::kan ^R^ ura4-D18 leu1-32* This study

*ade6-M210* h^+^

KGY6541 *prp19-GFP::kan ^R^ ade6-M216 ura4-D18 leu1-32* h^-^ Lab stock

KGY6756 *cdc5-myc_13_::kan ^R^ dre4-myc_13_::kan ^R^ cwf7-HA_3_::Kan^R^* This study

*ade6-M216 ura4-D18 leu1-32* h?

KGY6819 *dre4-TAP::kan ^R^ ade6-M210 ura4-D18 leu1-32* h^-^ This study

KGY6907 *saf1-flag_3_::kan ^R^ ade6-M210 ura4-D18 leu1-32* h^-^ This study

KGY6909 *saf3-TAP::kan ^R^ ade6-M210 ura4-D18 leu1-32* h^-^ This study

KGY6912 *saf3-GFP::kan ^R^ ade6-M210 ura4-D18 leu1-32* h^-^ This study

KGY7003 *prp19-TAP::kan ^R^ ade6-M210 ura4-D18 leu1-32* h^-^ This study

KGY7007 *saf1-flag_3_::kan ^R^ dre4-GFP::kan ^R^* h^-^  This study

*ade6-M21X ura4-D18 leu1-32*

KGY7041 *prp19-TAP::kan ^R^ cdc5-myc_13_::kan ^R^ dre4-GFP::kan ^R^* h^-^ This study

*ade6-M21X ura4-D18 leu1-32*

KGY7042 *prp19-TAP::kan ^R^ cwf2-myc_13_::kan ^R^ dre4-GFP::kan ^R^* h^-^ This study

*ade6-M21X ura4-D18 leu1-32*

KGY7043 *prp19-TAP::kan ^R^ prp17-myc_13_::kan ^R^ dre4-GFP::kan ^R^* h^+^ This study *ade6-M21X ura4-D18 leu1-32*

KGY7044 *saf1::ura4^+^ ade6-M21X ura4-D18 leu1-32* h^-^ This study

KGY7060 *saf3^+^/saf3::ura4^+^ade6-M210/ade6-M216 ura4-D18/ura4-D18* This study

*leu1-32/leu1-32* h^+^/h^-^

KGY7187 *prp19-TAP::kan ^R^ prp17-myc_13_::kan ^R^ cwf2-GFP::Kan ^R^* This study

*ade6-M210 ura4-D18 leu1-32* h^+^

KGY7212 *prp2-1 ura4-D18 leu1-32* h^-^Lab stock

KGY7577 *saf2-TAP::kan ^R^ ade6-M216 ura4-D18 leu1-32* h^-^ This study

KGY7922 *cwf2-TAP::kan ^R^ ade6-M210 ura4-D18 leu1-32* h^-^ Lab stock

KGY9141  *saf2-TAP::kan ^R^ prp19-myc_13_::kan ^R^* h^-^  This study

*ade6-M21X ura4-D18 leu1-32*

KGY9142  *saf1-flag_3_::kan ^R^ prp19-myc_13_::kan ^R^* h^-^  This study

*ade6-M21X ura4-D18 leu1-32*

KGY9143 *prp17-HA_3_-TAP::kan ^R^* *ade6-M216 ura4-D18 leu1-32* h^-^ This study

KGY9144 *dre4-HA_3_-TAP::kan ^R^ ade6-M216 ura4-D18 leu1-32* h^-^ This study

KGY9412 *saf2-GFP::kan ^R^ ade6-M210 ura4-D18 leu1-32* h^-^ This study

KGY9503 *saf2-GFP::kan ^R^ dre4-myc_13_::kan ^R^ ade6-M210 leu1-32* h^-^ This study

*ura4-D18*

KGY9742 *saf2^+^/saf2::ura4^+^ade6-M210/ade6-M216 ura4-D18/ura4-D18* This study

*leu1-32/leu1-32* h^+^/h^-^

KGY9921 *saf3-GFP::kan ^R^ prp19-myc_13_::kan ^R^ ade6-M21X leu1-32* h^-^ This study

*ura4-D18*

KGY10009 *saf3-GFP::kan ^R^ dre4-myc_13_::kan ^R^ ade6-M21X leu1-32* h^-^ This study

*ura4-D18*

KGY10032 *saf1-HA_3_-TAP::kan ^R^* h^-^ This study

KGY10033 *saf2-HA_3_-TAP::kan ^R^* h^-^ This study

KGY10034 *saf3-HA_3_-TAP::kan ^R^* h^-^ This study

KGY10188 *cwf11::ura4^+^* *saf1::ura4^+^ ade6-M21X ura4-D18 leu1-32* h^-^ This study

KGY10239 *cwf11::ura4^+^* *dre4::kan^R^ ade6-M21X ura4-D18 leu1-32* h^-^ This study

KGY11251 *byr4-linker-TAP*:: *kan^R^ ade6-M21X ura4-D18 leu1-32* h^-^ This study

***S. cerevisiae* strains**

KGY5311 *URN1-TAP_2_::His3MX6 MATa ura3Δ0 met15Δ0 leu2Δ0 his3Δ1* Open Biosystems KGY5754 *PRP17-GFP::Kan^R^ MATα, ura3-52, lys2-801, ade2-101,* This study

*leu2-Δ1, his3-Δ200*

KGY5755 *CWC2-GFP::Kan^R^ MATα, ura3-52, lys2-801, ade2-101,* This study

*leu2-Δ1, his3-Δ200*

KGY5757 *MIH1-GFP::Kan^R^ MATα, ura3-52, lys2-801, ade2-101,* This study

*leu2-Δ1, his3-Δ200*

KGY5758 *URN1-GFP::Kan^R^ MATα  ura3-52 lys2-801 ade2-101 leu2-Δ1* This study

*his3-Δ200*

KGY10056 *AIM4-myc_13_::kan^R^ MATa ura3-52 lys2-801 ade2-101leu2-∆1* This study

*trp1-∆1*

KGY10178 *AIM4-TAP_2_::HIS3MX6 MATa ura3Δ0 met15Δ0 leu2Δ0 his3Δ1* Open Biosystems

KGY10707 *CWC2-myc_13_::kan ^R^ URN1-GFP::kan ^R^* MAT? *ura3-52 lys2-801* This study

*ade2-101 leu2-Δ1, trp1-Δ1? his3-Δ200?*

KGY10127 *AIM4-myc::kan^R^ URN1-gfp::kanR MAT? ura3-52. lys2-801* This study

*ade2-101 leu2-Δ1, trp1-Δ1? his3-Δ200?*
