# Supplementary material for: Systematic Two-Hybrid and Comparative Proteomic Analyses Reveal Novel Yeast Pre-mRNA Splicing Factors Connected to Prp19
Source: PLoS One. 2011 Feb 28;6(2):e16719. doi: 10.1371/journal.pone.0016719 (PMC3046128; doi:10.1371/journal.pone.0016719)
Supplement: Table S2 — Heatmap of 100 most abundant proteins identified from Cwf2-, Prp17-, and Prp19- TAPs. “ORF” = open reading frame, “% Coverage” = % sequence coverage from MS analysis, “TSC” = total spectral counts, and shaded cells indicate protein abundance index (PAI, spectral counts/distinct peptides) numbers (Ref. 71) for the TAPs indicated at the top of each column. (PDF) [file pone.0016719.s009.pdf]

Table S2. Heatmap of 100 most abundant proteins identified from Cwf2-, Prp17-, and Prp19- TAPs

| ORF | Mol. Wt. | % Coverage | TSC | Protein | Cwf2 | Prp17 | Prp19 |
|---|---|---|---|---|---|---|---|
| SPAC4F8.12c | 274500 | 76 | 6055 | Spp42 | 13.25 | 14.81 | 11.98 |
| SPBC646.02 | 148300 | 76 | 3347 | Cwf11 | 12.97 | 14.31 | 14.83 |
| SPBC215.12 | 111100 | 73 | 2717 | Cwf10 | 14.92 | 16.04 | 11.32 |
| SPBC211.02c | 92600 | 84 | 2673 | Cwf3 | 13.55 | 16.21 | 12.87 |
| SPAC644.12 | 86800 | 82 | 2635 | Cdc5 | 17.85 | 15.39 | 8.58 |
| SPAC29A4.08c | 54100 | 77 | 2207 | Prp19/Cwf8 | 28.45 | 30.39 | 18.96 |
| SPBC6B1.10 | 63100 | 79 | 2049 | Prp17 | 17.67 | 17.78 | 14.58 |
| SPBC31F10.11c | 80800 | 76 | 1900 | Cwf4 | 11.98 | 16.76 | 9.69 |
| SPBP22H7.07 | 52400 | 83 | 1796 | Prp5 | 21.49 | 16.50 | 14.45 |
| SPBC1289.11 | 37400 | 92 | 1435 | Cwf17/Spf38 | 23.77 | 15.23 | 19.28 |
| SPCC188.11 | 62600 | 66 | 1277 | Prp45 | 15.31 | 16.89 | 10.47 |
| SPCC550.02c | 39500 | 81 | 1199 | Cwf5/Ecm2 | 14.97 | 16.80 | 11.38 |
| SPAC30D11.09 | 74400 | 71 | 1195 | Cwf19 | 8.22 | 12.39 | 6.87 |
| SPAC3A12.11c | 44200 | 81 | 1158 | Cwf2 | 18.18 | 16.48 | 11.22 |
| SPBC28F2.04c | 21300 | 82 | 823 | Cwf7 | 14.73 | 14.24 | 18.36 |
| SPBC337.06c | 30400 | 58 | 715 | Cwf15 | 12.47 | 26.40 | 6.60 |
| SPCP1E11.07c | 16600 | 65 | 610 | Cwf18 | 20.22 | 34.88 | 16.56 |
| SPBC1861.08c | 27200 | 87 | 588 | Lea1 | 12.40 | 12.14 | 8.50 |
| SPBC32F12.05c | 25600 | 80 | 549 | Cwf12 | 14.78 | 11.64 | 8.16 |
| SPBC19C2.14 | 11000 | 68 | 541 | Smd3 | 34.86 | 21.40 | 47.50 |
| SPBC24C6.11 | 17000 | 86 | 449 | Cwf14 | 17.07 | 10.33 | 9.75 |
| SPAC9.03c | 248800 | 49 | 448 | Brr2 | 2.11 | 3.32 | 3.84 |
| SPAC26A3.08 | 15400 | 95 | 397 | Smb1 | 11.38 | 6.22 | 14.85 |
| SPBC3E7.13c | 27900 | 70 | 391 | Syf2 Family | 3.85 | 7.50 | 7.92 |
| SPAC10F6.02c | 131400 | 56 | 371 | Prp22 | 2.11 | 3.29 | 4.48 |
| SPBC4B4.05 | 8600 | 94 | 366 | Smg1 | 16.90 | 19.00 | 9.22 |
| SPAC27D7.07c | 13000 | 57 | 318 | Smd1 | 34.25 | 21.25 | 24.00 |
| SPAC57A10.03 | 16800 | 50 | 315 | Cyp1 | 17.63 | 17.00 | 7.86 |
| SPCC364.02c | 43000 | 58 | 285 | Bis1 | 6.53 | 6.91 | 5.76 |
| SPBC11G11.06c | 9600 | 82 | 244 | Sme1 | 19.80 | 4.80 | 24.20 |
| SPBC3E7.14 | 8600 | 88 | 229 | Smf1 | 12.67 | 22.50 | 12.60 |
| SPBC428.12c | 12900 | 52 | 188 | RNA BP | 14.50 | 24.00 | 8.50 |
| SPBC8D2.09c | 12600 | 67 | 144 | Msl1 | 8.43 | 4.25 | 7.29 |
| SPAC19G12.07c | 69400 | 45 | 134 | Rsd1 | 3.38 | 3.36 | 1.50 |
| SPBC1711.17 | 132900 | 35 | 122 | Prp16 | 1.90 | 2.88 | 2.15 |
| SPAC20H4.06c | 60700 | 54 | 113 | RNA BP | 2.13 | 3.27 | 3.53 |
| SPAC4A8.09c | 34500 | 48 | 110 | Cwf21 | 2.45 | 5.22 | 3.60 |
| SPBC16H5.10c | 83800 | 39 | 97 | Prp43 | 3.00 | 4.18 | 3.00 |
| SPAC27F1.09c | 135100 | 30 | 93 | Prp10 | 2.00 | 3.00 | 3.57 |
| SPAC1F3.09 | 63200 | 38 | 78 | Mug16 | 1.50 | 2.79 | 4.13 |
| SPBC146.05c | 46000 | 44 | 74 | Cwf25 | 1.17 | 3.18 | 3.20 |
| SPAC20H4.09 | 73000 | 40 | 73 | RNA Helicase | 1.50 | 2.57 | 3.25 |
| SPAPJ698.03c | 134900 | 27 | 69 | Prp12 | 1.00 | 1.00 | 2.83 |
| SPAC9.13c | 31600 | 52 | 55 | Cwf16 | 0.00 | 5.29 | 2.00 |

| PAI |
|---|
| >20 |
| >15 |
| >10 |
| >7 |
| >4 |
| >2 |
| >0.5 |
| <0.5 |

| ORF | Mol. Wt. | % Coverage | TSC | Protein | Cwf2 | Prp17 | Prp19 |
|---|---|---|---|---|---|---|---|
| SPAC22A12.09c | 54400 | 37 | 53 | Sap11 | 2.00 | 1.00 | 4.55 |
| SPCC1620.10 | 36000 | 39 | 44 | Cwf26 | 1.00 | 5.00 | 2.17 |
| SPAC22F8.10c | 69100 | 36 | 43 | Sap14 | 2.00 | 1.00 | 2.23 |
| SPBC19C2.01 | 121200 | 23 | 42 | Cdc28 | 1.00 | 1.75 | 2.08 |
| SPAC22A12.16 | 53900 | 32 | 41 | ATP Synthase | 3.15 | 0.00 | 0.00 |
| SPAC1486.03c | 91600 | 17 | 40 | RNA BP | 1.00 | 2.60 | 2.17 |
| SPAC6G10.10c | 22300 | 37 | 40 | hHmmtag2 Homolog | 3.00 | 5.20 | 2.67 |
| SPBC36.09 | 57200 | 31 | 38 | Sap61 | 1.33 | 1.67 | 2.23 |
| SPBC20F10.01 | 20100 | 31 | 35 | Gar1 | 5.00 | 9.00 | 1.00 |
| SPBC530.14c | 61000 | 31 | 34 | Dsk1 | 0.00 | 2.67 | 2.00 |
| SPBC21C3.05 | 24900 | 59 | 33 | Sap62 | 1.67 | 1.33 | 4.00 |
| SPBC713.05 | 32900 | 24 | 32 | hMAPK Organizer 1 | 2.00 | 2.33 | 3.50 |
| SPAC24H6.04 | 53500 | 40 | 30 | Hxk1 | 2.00 | 0.00 | 0.00 |
| SPAC31G5.18c | 29200 | 42 | 30 | hC1ORF55 Related | 1.50 | 2.75 | 3.20 |
| SPAC1006.07 | 44400 | 30 | 29 | eIF4A | 2.42 | 0.00 | 0.00 |
| SPBC13E7.02 | 60700 | 23 | 29 | Cwf24 | 0.00 | 2.00 | 3.00 |
| SPAC26H5.10c | 17100 | 29 | 26 | Tif51 | 6.50 | 0.00 | 0.00 |
| SPBC1703.07 | 67200 | 21 | 26 | ATP Synthase | 2.60 | 0.00 | 0.00 |
| SPAC26F1.03 | 45100 | 38 | 24 | Pda1 | 2.00 | 0.00 | 0.00 |
| SPCC5E4.10c | 12000 | 49 | 23 |  | 2.00 | 4.50 | 4.00 |
| SPAC29A4.15 | 51600 | 30 | 20 | Srs1 | 1.82 | 0.00 | 0.00 |
| SPAC343.17c | 63600 | 15 | 18 | WDR70 Family | 0.00 | 2.33 | 1.33 |
| SPCC794.07 | 52000 | 19 | 18 | Acetyltransferase | 3.40 | 1.00 | 0.00 |
| SPBC146.07 | 58900 | 15 | 17 | Prp2 | 2.75 | 1.67 | 1.00 |
| SPAC17G6.14c | 49200 | 21 | 16 | Uap56 | 2.29 | 0.00 | 0.00 |
| SPAC56F8.05c | 31700 | 43 | 16 | Mug64 | 1.88 | 1.00 | 0.00 |
| SPAC31G5.01 | 35900 | 19 | 15 | Sap49 | 1.00 | 3.50 | 1.50 |
| SPBC428.02c | 42500 | 29 | 15 | Eca39 | 1.88 | 0.00 | 0.00 |
| SPAC1420.02c | 59300 | 23 | 13 | Cct5 | 1.30 | 0.00 | 0.00 |
| SPAC6F12.13c | 39500 | 20 | 13 | Fps1 | 2.17 | 0.00 | 0.00 |
| SPBC1289.03c | 24500 | 20 | 13 | Spi1 | 3.25 | 0.00 | 0.00 |
| SPBC4B4.09 | 71200 | 18 | 13 | Usp10 | 0.00 | 1.00 | 1.83 |
| SPBC646.09c | 57100 | 21 | 13 | Int6 | 1.86 | 0.00 | 0.00 |
| SPBP23A10.12 | 27600 | 13 | 13 | FRG1 Family | 0.00 | 3.33 | 3.00 |
| SPCC1020.06c | 35200 | 24 | 13 | Tal1 | 2.17 | 0.00 | 0.00 |
| SPCC1442.09 | 54900 | 16 | 13 | Trp3 | 2.17 | 0.00 | 0.00 |
| SPCC16A11.13 | 30900 | 23 | 13 | Usp10 | 1.67 | 3.00 | 1.67 |
| SPCC736.15 | 39800 | 22 | 13 | Kinase Inhibitor | 2.17 | 0.00 | 0.00 |
| SPCC962.06c | 63600 | 7 | 13 | Bpb1 | 2.33 | 2.00 | 2.00 |
| SPBC29A3.07c | 13100 | 36 | 12 | Sf3B14 Homolog | 2.00 | 0.00 | 6.00 |
| SPBC342.02 | 92000 | 9 | 12 | Qrs1 | 1.50 | 0.00 | 0.00 |
| SPAC13A11.02c | 56300 | 20 | 11 | Erg11 | 1.75 | 0.00 | 1.33 |
| SPAC19A8.10 | 28900 | 7 | 11 | Rfp1 | 5.00 | 1.00 | 0.00 |
| SPAC1D4.04 | 56600 | 13 | 11 | Cct2 | 1.83 | 0.00 | 0.00 |
| SPAC24H6.10c | 39700 | 21 | 11 | Aldolase | 1.83 | 0.00 | 0.00 |
| SPAC6F12.10c | 144800 | 9 | 11 | Ade3 | 1.38 | 0.00 | 0.00 |

| PAI |
|---|
| >20 |
| >15 |
| >10 |
| >7 |
| >4 |
| >2 |
| >0.5 |
| <0.5 |

| ORF | Mol. Wt. | % Coverage | TSC | Protein | Cwf2 | Prp17 | Prp19 |
|---|---|---|---|---|---|---|---|
| SPBC1703.10 | 22800 | 39 | 11 | Ypt1 | 1.57 | 0.00 | 0.00 |
| SPBC211.05 | 9500 | 60 | 11 | Splicing factor 3B | 0.00 | 1.00 | 3.33 |
| SPAC25G10.01 | 33500 | 9 | 10 | RNA BP | 1.67 | 5.00 | 0.00 |
| SPBC12C2.06 | 56300 | 16 | 10 | Dbp5 | 1.67 | 0.00 | 0.00 |
| SPBC13G1.02 | 46000 | 14 | 10 | Guanyltransferase | 2.00 | 0.00 | 0.00 |
| SPBC30D10.13c | 39600 | 16 | 10 | Pdb1 | 3.33 | 0.00 | 0.00 |
| SPAC13G7.06 | 30500 | 34 | 9 | Met16 | 1.50 | 0.00 | 0.00 |
| SPAC1F12.07 | 42700 | 13 | 9 | Aminotransferase | 2.25 | 0.00 | 0.00 |
| SPAC23H3.02c | 13200 | 46 | 9 | Ini1 | 1.00 | 2.00 | 2.00 |

| PAI |
|---|
| >20 |
| >15 |
| >10 |
| >7 |
| >4 |
| >2 |
| >0.5 |
| <0.5 |

"ORF" = open reading frame, "% Coverage" = % sequence coverage from MS analysis, "TSC" = total spectral counts, and shaded cells indicate protein abundance index (PAI, spectral counts/distinct peptides) numbers (Ref. 71) for the TAPs indicated at the top of each column.
