# Supplementary material for: Systematic Two-Hybrid and Comparative Proteomic Analyses Reveal Novel Yeast Pre-mRNA Splicing Factors Connected to Prp19
Source: PLoS One. 2011 Feb 28;6(2):e16719. doi: 10.1371/journal.pone.0016719 (PMC3046128; doi:10.1371/journal.pone.0016719)
Supplement: Table S3 — Heatmap of splicing-associated proteins identified from Dre4-, Saf1-, Saf2-, and Saf3- TAPs. “ORF” = open reading frame, “% Coverage” = % sequence coverage from MS analysis, “TSC” = total spectral counts, and shaded cells indicate protein abundance index (PAI, spectral counts/distinct peptides) numbers (Ref. 71) for the TAPs indicated at the top of each column. (PDF) [file pone.0016719.s010.pdf]

Table S3. Heatmap of splicing-associated proteins identified from Dre4-, Saf1-, Saf2-, and Saf3- TAPs

ORF	% Coverage	Mol. Wt.	TSC	Protein	Dre4	Saf1	Saf2	Saf3
SPAC1782.03	50	40700	814	Saf3	2.91	0.00	2.00	26.90
SPAC13C5.02	75	48500	548	Dre4	13.08	7.50	0.00	4.00
SPAC22F3.11c	71	20100	405	Snu23	2.20	0.00	0.00	28.14
SPCC663.11	64	32300	397	Saf1	12.09	11.91	0.00	0.00
SPAC4F8.12c	35	274500	283	Spp42	2.58	3.00	2.09	2.92
SPBC19C2.08	72	24400	258	Prp38	2.25	0.00	0.00	19.15
SPBC215.12	44	111100	174	Cwf10	2.96	3.50	1.67	4.39
SPAC9.03c	27	248800	151	Brr2	1.86	0.00	1.00	2.72
SPAPJ698.03c	26	134900	90	Prp12	2.08	0.00	2.00	2.24
SPBC36.09	35	57200	86	Sap61	2.43	0.00	2.40	3.08
SPAC27F1.09c	30	135100	84	Prp10	2.11	0.00	1.00	2.87
SPAC22F8.10c	36	69100	78	Sap14	2.53	0.00	2.25	3.10
SPAC22A12.09c	44	54400	65	Sap11	2.88	0.00	1.50	2.60
SPBC1289.11	39	37400	60	Cwf17/Spf38	1.83	9.00	2.75	5.00
SPAC29A4.08c	34	54100	56	Prp19/Cwf8	1.88	4.33	3.40	2.75
SPBC21C3.05	51	24900	52	Sap62	3.00	0.00	4.00	3.38
SPAC19G12.07c	26	69400	49	Rsd1	2.50	0.00	1.00	13.00
SPBC4B4.05	64	8600	44	Smg1	1.67	13.50	3.00	4.50
SPAC2F3.14c	52	37600	44	Saf2	3.00	0.00	3.00	5.00
SPBP22H7.07	16	52400	42	Prp5	2.50	16.50	2.00	2.00
SPBC6B1.07	19	103000	42	Prp1	0.00	0.00	1.00	2.73
SPCC1620.10	43	36000	39	Cwf26	3.00	0.00	1.67	2.00
SPAC56F8.05c	42	31700	38	Mug64	1.50	4.40	1.22	1.00
SPAC644.12	19	86800	32	Cdc5	1.00	4.80	1.25	1.00
SPBC1861.08c	54	27200	31	Lea1	1.88	0.00	1.00	2.33
SPBC28F2.04c	32	21300	25	Cwf7	1.00	19.00	1.33	1.00
SPBC646.02	6	148300	24	Cwf11	1.50	10.00	1.00	0.00
SPCC188.11	23	62600	24	Prp45	1.29	0.00	2.25	2.00
SPAC227.12	20	51700	23	Prp4	0.00	0.00	0.00	2.88
SPBC211.02c	11	92600	21	Cwf3	1.60	5.00	1.00	1.00
SPBC146.07	15	58900	21	Prp2	1.80	0.00	3.33	2.00
SPBC29A3.07c	57	13100	21	Sfb314 Homolog	2.00	0.00	0.00	2.75
SPAC26A3.08	46	15400	19	Smb1	4.00	2.00	0.00	3.67
SPAC167.03c	18	74700	19	Snu66	0.00	0.00	0.00	2.71
SPAC27D7.07c	39	13000	18	Smd1	3.00	0.00	0.00	3.00
SPBC6B1.10	22	63100	15	Prp17	1.50	2.50	1.33	0.00
SPBC119.13c	14	57700	15	Prp31	0.00	0.00	0.00	2.50
SPAC29E6.02	11	62500	13	Prp3	0.00	0.00	0.00	2.17
SPAC3A12.11c	22	44200	12	Cwf2	1.50	0.00	2.00	0.00
SPBC19C2.14	55	11000	12	Smd3	2.00	0.00	0.00	2.00
SPBC31F10.11c	11	80800	12	Cwf4	1.80	0.00	1.00	2.00
SPBC887.05c	21	25300	9	Cwf29	2.67	0.00	0.00	1.00
SPCC550.02c	20	39500	9	Cwf5/Ecm2	1.00	2.00	2.00	2.00
SPBC337.06c	26	30400	8	Cwf15	2.00	2.00	2.00	0.00
SPCC162.01c	14	29300	5	tri-snRNP subunit	0.00	0.00	1.67	0.00
SPCC1281.02c	13	34600	4	Spf30	0.00	0.00	0.00	1.33

PAI
>20
>15
>10
>7
>4
>2
>0.5

ORF = open reading frame, "% Coverage" = % sequence coverage from MS analysis, "TSC" = total spectral counts, and shaded cells indicate protein abundance index (PAI, spectral counts/distinct peptides) numbers (Ref. 71) for the TAPs indicated at the top of each column.
