# Supplementary material for: Systematic Two-Hybrid and Comparative Proteomic Analyses Reveal Novel Yeast Pre-mRNA Splicing Factors Connected to Prp19
Source: PLoS One. 2011 Feb 28;6(2):e16719. doi: 10.1371/journal.pone.0016719 (PMC3046128; doi:10.1371/journal.pone.0016719)
Supplement: Table S4 — Splicing-related proteins identified from Aim4- and Urn1- TAPs. “ORF” = open reading frame, “% Coverage” = % sequence coverage from MS analysis, “TSC” = total spectral counts, and shaded cells indicate protein abundance index (PAI, spectral counts/distinct peptides) numbers (Ref. 71) for the TAPs indicated at the top of each column. (PDF) [file pone.0016719.s011.pdf]

Table S4. Splicing-related proteins identified from Aim4- and Urn1- TAPs

ORF	Mol. Wt.	% Coverage	TSC	Protein	Aim4	Urn1
YPR152C	54138	71	3770	Urn1	34.55	40.18
YBR194W	14234	87	457	Aim4	17.21	14.40
YLL036C	56569	52	264	Prp19	6.11	6.16
YMR213W	67730	60	103	Cef1	2.07	3.70
YDL014W	34465	48	77	Nop1	0.00	5.13
YAL032C	42482	49	76	Prp45	2.00	3.44
YPL151C	50700	45	75	Prp46	1.60	3.47
YNL112W	60999	28	71	Dbp2	4.36	2.88
YML010W	115649	26	60	Spt5	2.21	2.07
YOR310C	56956	35	53	Nop58	2.00	3.64
YHR165C	279501	11	48	Prp8	1.00	2.42
YPR101W	20709	70	46	Snt309	2.00	3.45
YLR117C	82444	27	42	Clf1	1.33	2.53
YNL088W	164213	15	40	Top2	0.00	2.11
YDR432W	45407	18	37	Npl3	5.75	3.50
YER172C	246183	8	35	Brr2	2.67	1.69
YML017W	65584	28	33	Psp2	1.40	3.25
YKL173W	114040	13	30	Snu114	1.00	2.45
YHR121W	21313	42	28	Lsm12	2.33	3.00
YML049C	153783	13	28	Rse1	1.60	1.82
YMR288W	110027	16	26	Hsh155	1.00	2.18
YLR275W	12853	40	25	Smd2	1.00	5.75
YNL248C	46650	33	23	Rpa49	0.00	2.30
YMR229C	193133	9	23	Rrp5	0.00	1.92
YER029C	22379	37	21	Smb1	1.00	3.33
YAL059W	23935	26	18	Ecm1	3.33	2.67
YMR240C	50253	15	17	Cus1	2.00	2.50
YOL139C	24254	40	17	Cdc33	2.00	1.83
YCR030C	96137	8	15	Syp1	1.57	2.00
YLR196W	63803	15	15	Pwp1	2.00	1.83
YPL032C	92143	12	15	Svl3	2.00	1.75
YBR188C	15970	24	13	Ntc20	2.00	2.33
YPL070W	69538	16	13	Muk1	1.25	1.33
YEL015W	61340	15	12	Edc3	1.00	1.83
YOR319W	24503	30	12	Hsh49	3.00	1.50
YPL213W	27193	37	11	Lea1	2.00	1.80
YMR128W	144953	9	11	Ecm16	0.00	1.10
YDR299W	61203	7	10	Bfr2	1.50	1.75
YDL030W	63029	17	10	Prp9	0.00	1.67

PAI
>20
>15
>10
>7
>4
>2
>0.5
<0.5

ORF = open reading frame, "% Coverage" = % sequence coverage from MS analysis, "TSC" = total spectral counts, and shaded cells indicate protein abundance index (PAI, spectral counts/distinct peptides) numbers (Ref. 71) for the TAPs indicated at the top of each column.
