# Supplementary material for: Systematic Two-Hybrid and Comparative Proteomic Analyses Reveal Novel Yeast Pre-mRNA Splicing Factors Connected to Prp19
Source: PLoS One. 2011 Feb 28;6(2):e16719. doi: 10.1371/journal.pone.0016719 (PMC3046128; doi:10.1371/journal.pone.0016719)
Supplement: Table S5 — Heatmap of other (non-splicing) proteins identified from Aim4- and Urn1- TAPs. “ORF” = open reading frame, “% Coverage” = % sequence coverage from MS analysis, “TSC” = total spectral counts, and shaded cells indicate protein abundance index (PAI, spectral counts/distinct peptides) numbers (Ref. 71) for the TAPs indicated at the top of each column. (PDF) [file pone.0016719.s012.pdf]

Table S5. Heatmap of other (non-splicing) proteins identified from Aim4- and Urn1- TAPs

ORF	Mol. Wt.	% Coverage	TSC	Protein	Aim4	Urn1
YNL067W	21657	57	132	Rpl9B	9.86	6.30
YGL030W	11415	76	94	Rpl30	7.60	9.33
YHR174W	46914	58	80	Eno2	3.25	3.86
YER151C	101916	29	77	Ubp3	2.87	2.83
YBR009C	11368	58	76	Hhf1	3.50	8.86
YGR148C	17547	28	72	Rpl24B	3.25	8.43
YLR197W	56864	43	68	Nop56	2.00	3.88
YBR127C	57749	57	67	Vma2	2.00	2.61
YGL031C	17613	28	63	Rpl24A	3.67	7.43
YGR254W	46816	40	62	Eno1	3.86	3.89
YLR432W	56584	33	57	Imd3	3.44	2.89
YDR450W	17037	58	55	Rps18A	5.25	3.78
YCR012W	44738	48	54	Pgk1	2.40	2.80
YOL086C	36849	39	52	Adh1	2.67	3.00
YBL072C	22489	47	52	Rps8A	6.00	2.44
YPL131W	33714	30	48	Rpl5	4.80	4.80
YJL130C	245124	11	48	Ura2	1.80	2.00
YGR162W	107101	17	47	Tif4631	2.14	2.91
YBR189W	22298	42	45	Rps9B	2.14	4.29
YGR132C	31427	43	44	Phb1	3.17	3.57
YKL182W	228689	10	44	Fas1	2.27	1.90
YOR153W	170437	13	42	Pdr5	1.43	2.13
YML056C	56394	26	41	Imd4	3.25	2.50
YKL152C	27608	40	39	Gpm1	2.67	3.88
YLR044C	61495	38	39	Pdc1	1.80	2.50
YHR216W	56530	24	38	Imd2	2.88	3.00
YMR186W	80899	19	37	Hsc82	2.25	1.90
YGR097W	126863	24	37	Ask10	1.00	1.75
YNL138W	57521	28	36	Srv2	2.11	1.89
YKL060C	39620	27	34	Fba1	4.50	3.57
YGR086C	38349	27	34	Pil1	3.25	3.50
YAL035W	112268	15	34	Fun12	2.00	2.46
YGR118W	16038	54	32	Rps23A	2.00	6.00
YGL103W	16722	33	31	Rpl28	2.67	5.75
YGR186W	82194	14	31	Tfg1	2.83	2.00
YHL015W	13907	52	29	Rps20	3.50	2.00
YDR483W	51386	23	27	Kre2	2.50	2.40
YKL081W	46520	17	27	Tef4	3.33	2.33
YDR190C	50453	27	27	Rvb1	1.80	1.80
YIL022W	48854	29	27	Tim44	2.25	1.64
YGL049C	103898	10	26	Tif4632	1.33	4.40
YBR169C	77620	9	26	Sse2	1.80	4.25
YKL054C	83973	20	26	Def1	3.00	3.33
YGR130C	92698	18	26		2.33	1.50

PAI
>20
>15
>10
>7
>4
>2
>0.5
<0.5

ORF	Mol. Wt.	% Coverage	TSC	Protein	Aim4	Urn1
YBL030C	34426	29	25	Pet9	1.80	2.67
YBR079C	110343	14	25	Rpg1	2.50	1.67
YKL022C	94991	12	25	Cdc16	2.50	1.67
YLL013C	98067	17	25	Puf3	1.50	1.60
YPL004C	38071	22	24	Lsp1	2.83	3.50
YDR172W	76551	19	24	Sup35	2.50	2.00
YPL085W	241694	9	24	Sec16	1.60	1.78
YGL013C	121793	12	24	Pdr1	1.67	1.58
YDR379W	113290	13	24	Rga2	1.56	1.43
YPL231W	206945	9	23	Fas2	1.50	1.57
YMR072W	21562	42	22	Abf2	1.00	2.86
YDR050C	26795	32	22	Tpi1	1.67	2.40
YMR205C	104617	12	22	Pfk2	2.00	2.00
YER006W	57708	24	22	Nug1	1.67	1.89
YPL235W	51611	28	22	Rvb2	2.00	1.25
YJL076W	128530	13	21	Net1	0.00	1.75
YGR240C	107969	16	21	Pfk1	1.50	1.29
YLR180W	41818	14	20	Sam1	2.00	3.20
YIR006C	160267	13	20	Pan1	1.42	1.00
YFL016C	55561	24	19	Mdj1	0.00	2.38
YCR009C	30250	30	19	Rvs161	3.00	2.33
YDL185W	118636	10	19	Tfp1	2.60	1.50
YDR394W	47893	18	18	Rpt3	2.67	2.50
YJL005W	227832	5	18	Cyr1	2.00	1.50
YKR006C	30271	37	17	Mrpl13	1.33	3.25
YDR293C	139953	9	17	Ssd1	2.20	2.00
YKL145W	51982	22	17	Rpt1	1.50	1.83
YMR012W	145164	8	17	Clu1	1.33	1.63
YGL106W	16444	36	17	Mlc1	2.80	1.50
YPR024W	81771	14	17	Yme1	2.40	1.25
YIL148W	14554	37	16	Rpl40	1.50	2.60
YLR342W	214849	5	16	Fks1	1.40	2.25
YLR167W	17216	30	16	Rps31	1.67	2.20
YIL105C	77995	19	16	Slm1	1.50	1.63
YKR029C	85479	11	15	Set3	2.00	2.17
YMR146C	38755	20	15	Tif34	2.00	1.00
YGL245W	80842	9	15	Gus1	2.33	1.00
YOR361C	88129	12	14	Prt1	1.25	1.80
YMR246W	77267	8	14	Faa4	2.25	1.67
YNR016C	250351	4	14	Acc1	1.20	1.60
YER177W	30091	41	14	Bmh1	1.00	1.57
YPR091C	87323	14	14		1.38	1.00
YCL030C	87721	11	14	His4	1.83	1.00
YDR229W	49975	16	13	Ivy1	1.67	2.67
YKR003W	51588	10	13	Osh6	2.00	1.75
YCR077C	88495	12	13	Pat1	1.67	1.60

PAI
>20
>15
>10
>7
>4
>2
>0.5
<0.5

ORF	Mol. Wt.	% Coverage	TSC	Protein	Aim4	Urn1
YNL172W	196142	5	13	Apc1	1.83	1.00
YDL126C	91995	12	13	Cdc48	2.00	1.00
YDR289C	46488	18	13	Rtt103	2.00	1.00
YOR227W	139456	6	12	Her1	1.60	4.00
YGL068W	20650	18	12	Mnp1	1.25	3.50
YDR310C	118200	10	12	Sum1	1.00	2.20
YLR175W	54704	18	12	Cbf5	2.00	1.67
YPL237W	31574	19	12	Sui3	2.25	1.50
YJL095W	164194	5	12	Bck1	1.33	1.33
YML072C	171074	5	11	Tcb3	1.00	2.25
YLL026W	102034	10	11	Hsp104	1.50	2.00
YJL122W	19287	34	11	Alb1	1.67	2.00
YBR142W	87047	9	11	Mak5	1.00	2.00
YCR033W	138396	7	11	Snt1	0.00	1.83
YHR020W	77386	11	11		2.00	1.25
YPL226W	134330	11	11	New1	1.40	1.00
YMR315W	38215	18	10		1.50	2.00
YGL099W	72727	9	10	Lsg1	1.50	1.75
YIL035C	44667	11	10	Cka1	1.25	1.67
YJR121W	54793	15	10	Atp2	1.67	1.67
YHR084W	77866	11	10	Ste12	0.00	1.67
YGR090W	140483	6	10	Utp22	0.00	1.43
YIL053W	27947	24	10	Rhr2	1.67	1.25
YAR002W	59039	10	10	Nup60	2.00	1.00
YOR136W	39739	22	10	Idh2	1.67	0.00
YBR049C	91874	7	9	Reb1	1.00	1.75
YGR282C	34118	20	9	Bgl2	1.50	1.50
YLR072W	78212	7	9		1.50	1.50
YHR099W	433175	2	9	Tra1	1.00	1.40
YML007W	72532	12	9	Yap1	1.25	1.33
YGR005C	46605	16	8	Tfg2	1.33	1.33
YNL271C	219701	3	8	Bni1	1.50	1.25
YDR074W	102976	7	8	Tps2	1.20	1.00
YOL016C	50447	12	8	Cmk2	1.20	1.00
YDL140C	191610	2	8	Rpo21	1.67	1.00
YJL176C	92926	9	7	Swi3	1.25	1.00

PAI
>20
>15
>10
>7
>4
>2
>0.5
<0.5

ORF = open reading frame, "% Coverage" = % sequence coverage from MS analysis, "TSC" = total spectral counts, and shaded cells indicate protein abundance index (PAI, spectral counts/distinct peptides) numbers (Ref. 71) for the TAPs indicated at the top of each column.
